# Analysis, Occurrence and Exposure Evaluation of Antibiotic and Anthelmintic Residues in Whole Cow Milk from China

**DOI:** 10.3390/antibiotics12071125

**Published:** 2023-06-29

**Authors:** Liming Chang, Sishi Du, Xiaojiao Wu, Jian Zhang, Zhiwei Gan

**Affiliations:** 1 Department of Pharmacy, West China Hospital, Sichuan University, Chengdu 610041, China; changliming@wchscu.cn (L.C.); dusishi@wchscu.cn (S.D.); wuxiaojiao@wchscu.cn (X.W.); 2 College of Architecture and Environment, Sichuan University, Chengdu 610065, China; ganzhiwei@scu.edu.cn

**Keywords:** QuEChERS, whole cow milk, antibiotics, anthelmintics, exposure

## Abstract

An optimized QuEChERS method for the simultaneous extraction of 26 antibiotics and 19 anthelmintics in whole cow milk was established, followed by UHPLC-MS/MS analysis. Briefly, 20 mL acetonitrile with 1 g disodium hydrogen citrate, 2 g sodium citrate, 4 g anhydrous MgSO_4_, and 1 g sodium chloride were added to 10 g milk for target chemical extraction, followed by 50 mg anhydrous MgSO_4_ for purification. Satisfactory recoveries were obtained using the modified QuEChERS method, with recoveries of the antibiotics ranging from 79.7 to 117.2%, with the exception of norfloxacin, which was at 53.4%, while those for anthelmintics were in the range of 73.1–105.1%. The optimized QuEChERS method presented good precision, with relative standard deviations ranging from 7.2 to 18.6% for both antibiotics and anthelmintics. The method was successfully applied to analyze the antibiotics and anthelmintics in 56 whole cow milk samples from China. Briefly, the detection frequencies and concentrations of most of the antibiotics and anthelmintics were low in the whole cow milk samples, with concentrations ranging from below LOD to 4296.8 ng/kg. Fenbendazole, febantel, enrofloxacin, levofloxacin, sulfadiazine, and sulfamethoxazole were the predominant drug residues in the whole cow milk samples. Spatial distribution was found for those antibiotics and anthelmintics with detection frequency higher than 50%, especially for the antibiotics, indicating regional differences in drug application. Based on the current study, exposure to antibiotics and anthelmintics through whole cow milk consumption are lower than the acceptable daily intake values suggested by the China Institute of Veterinary Drug Control. However, long-term exposure to low doses of antibiotics and anthelmintics still needs attention and merits further study.

## 1. Introduction

Anthelmintics and antibiotics, part of the larger sector of animal pharmaceuticals, are widely used for the prevention and control of parasitic and bacterial diseases in agriculture and aquaculture in the world [1,2,3,4]. Some of them have also found applications as pre- or post-harvest fungicides for crops, fruits, and vegetables [2]. In 2014, anthelmintic sales totaled RMB 1.725 billion in China, accounting for 10.15% of the market share of chemical medicine preparations [5], while annual antibiotic consumption in China reached 162,000 tons in 2013, accounting for 50% of global usage [6]. Since the therapeutic effect of the individual drug is limited, the combination use of anthelmintic and antibiotic products is required to control mixed helminthic and bacterial infections [7]. Owing to the lipophilic properties and incomplete metabolism, anthelmintics and antibiotics used in lactating cows can cause potentially harmful concentrations of substances to remain in their body and milk [8,9,10]. Some drugs are associated with several toxic effects like teratogenesis and embryotoxicity [1,11,12]. Potential harmful consequences also include allergic reactions, liver damage, yellow teeth, gastrointestinal disturbances, as well as an increase in pathogens resistant to antimicrobial agents [13,14]. Therefore, in the European Union (EU), abamectin (ABA), ivermectin (IVE), and doramectin (DOR) are banned for use on lactating animals, and the maximum residue limits (MRLs) in milk for some antibiotics have also been set, such as 10 μg/kg for fenbendazol (FEN) and 100 μg/kg for enrofloxacin (ENR) in the Commission Regulation (EU) No. 37/2010 [15].

Food safety caused by these two kinds of drug residues has aroused public concern. Liu et al. [16] found that the residues of cefquinome and spiramycin in milk samples from China were 2 and 30 µg/kg, respectively. Azithromycin (9708.7 μg/kg) and tetracycline (5460 μg/kg) were detected in milk samples collected from Karnataka, India [17]. The drug residues could enter the human body through milk consumption and might cause health risks for humans.

Traditional pretreatment methods, such as liquid–liquid extraction (LLE), solid-phase extraction (SPE), and matrix solid-phase dispersion (MSPD), are time-consuming and laborious, and their application scope is not wide. As a new pretreatment method, QuEChERS (quick, easy, cheap, effective, rugged, safe) has the characteristics of being fast, simple, cheap, effective, stable, and safe. In addition, it does not require glassware or ancillary equipment, uses low volumes of solvent, generates little solvent waste, and provides high recovery of analytes [18]. There are several studies regarding the anthelmintic or antibiotic analysis in milk, but most focus on only a few compounds or one class of drugs [19,20]. China is the world’s third largest milk producer, after India and the United States [21]. In 2019, China’s total milk output reached 32.01 million tons [22]. Therefore, it is necessary to establish a rapid, efficient, simple, and economical method to detect multiple classes of anthelmintics and antibiotics in milk simultaneously, in order to ensure the safety of milk.

Therefore, the aims of this study were to establish a QuEChERS method for simultaneously extracting 19 kinds of anthelmintics and 26 kinds of antibiotics from whole cow milk samples at the same time to detect the drug residues in Chinese whole cow milk samples using the developed UHPLC-MS/MS method in the previous studies coupled with our established pretreatment method in this study [23,24], and to evaluate daily intakes of the anthelmintics and antibiotics of Chinese people via whole cow milk consumption. To carry this out, samples of whole cow milk were collected from online markets of 21 provinces, 3 municipalities directly under the central government, and 4 autonomous regions in China, which can represent the characteristics of local whole cow milk and reflect the current situation of the dairy industry in China. To the best of our knowledge, this is the first systemic study to simultaneously evaluate seven classes of anthelmintic residues in Chinese whole cow milk samples.

## 2. Materials and Methods

### 2.1. Chemicals and Reagents

Detailed information on the investigated 26 antibiotics and 19 anthelmintics are given in Table S1 in the Supplementary Materials (SM). The sources of the reagents used in this study are shown as follows: the antibiotic and anthelmintic standards (Dr. Ehrenstorfer), the ISs (Dr. Ehrenstorfer, Toronto Research Chemicals, and Witega Laboratorien), ammonium formate, formic acid, acetic acid, HPLC-grade methanol and acetonitrile, and graphitized carbon black (GCB) (CNW Technologies, Duesseldorf, Germany), anhydrous magnesium sulfate (anhydrous MgSO_4_) (Thermo Fisher Scientific, Waltham, MA, USA), other chemical reagents like sodium acetate (C_2_H_3_NaO_2_), sodium chloride (NaCl), sodium citrate (SCTD), disodium hydrogen citrate (SCDS), and ammonium chloride (NH_4_Cl) (Sigma-Aldrich, St. Louis, MO, USA), and primary-secondary amine (PSA) and C18 (Agilent Technologies, Inc., Santa Clara, CA, USA). Milli-Q water was used throughout the study.

### 2.2. Sample Collection

A total of 56 different pure whole cow milk samples produced locally from 21 provinces, 3 municipalities directly under the central government, and 4 autonomous regions were purchased from online businesses. All of the whole cow milk samples were carefully examined to ensure that the source of milk was in the corresponding province; the milk samples investigated in this study were all from the top one or three brands in the local market, and the details are given in Table S2 in the SM. The samples were preserved at 4 °C until analysis.

### 2.3. Extraction Optimization

Four different versions of QuEChERS methods were evaluated in this study, including acetate-buffered version (V1), unbuffered version (V2), citrate-buffered version (V3), and ammonium version (V4) (Table 1). Briefly, approximately 10.0 g of whole cow milk was weighed in a 50 mL polypropylene (PP) centrifuge tube, followed by spiking of mixed antibiotic and mixed anthelmintic IS solution of 10 mg/L. The PP tube was vortexed for 2 min and let stand for 20 min. In the case of V1, 10/15/20 mL of acetonitrile containing, respectively, 0.5/1/2/3% acetic acid (*v*/*v*) was infused; subsequently, a mixture of sodium acetate and anhydrous MgSO_4_ (1:2/1:4/1:5, *w*:*w*) was added. For the V2, a portion of 10/15/20 mL acetonitrile containing a mixture of sodium acetate and anhydrous MgSO_4_ (1:2/1:4/1:5, *w*:*w*) was added. For the V3, acetonitrile containing a mixture of SCTD, SCDS, sodium chloride, and anhydrous MgSO_4_ (SCDS:SCTD = 1:2/1:1/2:1, *w*:*w*; sodium chloride:anhydrous MgSO_4_ = 1:2/1:4/1:5, *w*:*w*) was added, while for the V4, a mixture of ammonium chloride and anhydrous MgSO_4_ (1:2/1:4/1:5, *w*:*w*) was employed. After vortexing for 2 min, 1.0 mL of the supernatant (4000 r/min, 5 min) was collected into a 1.5 mL PP tube, and then 50/100/150/200 mg of anhydrous MgSO_4_ and 10/20/50/100 mg of PSA, 10/30/50 mg C18, or 10/30/50 mg GCB were added. The above-mentioned 1 mL extract was centrifuged at 15,000 r/min for 10 min after 2 min of vortexing. Finally, 0.5 mL of the supernatant was collected, and evaporated to dry under N_2_, and reconstituted to 0.2 mL with methanol–water (1:1, *v*:*v*) for UHPLC-MS/MS analysis.

### 2.4. Recovery and Matrix Effect

Post-extraction spike method was used to evaluate the recoveries and matrix effects (ME) of the QuEChERS method. The recovery and ME were calculated using the following equations:

Recovery = (*c_pre_*− *c_non_*)/(*c_post_*− *c_non_*) × 100% (1)

ME = (*c_post_*− *c_non_*)/*c_stan_* (2)

*c_pre_*: concentration of pre-spiked sample, µg/L.
*c_post_*: concentration of post-spiked sample, µg/L.
*c_non_*: concentration of non-spiked sample, µg/L.
*c_stan_*: concentration of standard solution.

In the case of pre-spiked samples, a mixture of standard solution of the antibiotics and anthelmintics was spiked into whole cow milk samples before extraction with 50 µL solution containing 10 µg·mL^−1^ of each analyte, while for post-spiked samples, the analytes were spiked after extraction at the same concentration as those of the corresponding pre-spiked samples.

### 2.5. Instrumental Analysis

The anthelmintics were analyzed using the method developed in our previous study by UHPLC-MS/MS (SCIEX, 4500QTrap, Redwood City, CA, USA) [24], while the analysis of the antibiotics was described by Hu et al. [23], and the details are given in Section S1 in the SM, and the detailed parameters are given in Table S3 in the SM. The limit of detection (LOD) and the limit of quantification (LOQ) are given in Table S4 in the SM.

### 2.6. Quality Control and Quality Assurance

Each whole cow milk sample was treated in duplicate, and the concentration was then taken as the average of the two. Blanks and a standard solution (10 µg·L^−1^) were run every 30 samples to check for carryover, background contamination, and accuracy. The target chemical compounds were below the LOD in the blanks. The LOD was defined as the spiked concentration with a signal-to-noise (S/N) ratio of 3, and the LOQ was defined as 10. The results of QA/QC are shown in Table S4. 

### 2.7. Estimated Daily Intakes of the Antibiotics and Anthelmintics via Whole Cow Milk Consumption

The estimated daily intakes (EDI) of antibiotics and anthelmintics via whole cow milk intake were calculated for three different age groups (toddlers: 2–5 years, teenagers: 6–17 years, and adults: >18 years) using the equation listed below:

D = C × 1.0288 × DC/BW(3)

where D (ng/kg/d) is the daily exposure amount of antibiotics and anthelmintics via milk consumption; C is the antibiotic and anthelmintic concentrations in the milk sample (ng/kg); 1.0288 is the density of milk (kg/L); DC is the daily consumption of milk (L/d); and BW is the average body weight (kg). All of the parameters for exposure evaluation used in this study are shown in Table S5 in the SM.

Two different scenarios (A and B) were employed to evaluate daily human exposure to antibiotics and anthelmintics via whole cow milk consumption. Briefly, median antibiotic and anthelmintic concentrations were used in Scenario A, which represented the mean exposure scenario. In comparison, 95th-percentile antibiotics and anthelmintic concentrations were used in Scenario B, which represented the high-exposure scenario. Compounds with detection frequencies lower than 50% did not take part in calculation.

### 2.8. Statistical Analysis

All of the statistical analysis was performed using SPSS 22.0. Compounds with detection frequencies lower than 50% did not take part in statistical analysis, and the concentrations below LODs and LOQs were replaced with 1/2 LODs and 1/2 LOQs, respectively. Non-parametric test (Mann–Whitney U test) was conducted to distinguish differences between two sets of data because of abnormal distribution of the data. Moreover, correlation test of two variables was implemented using Spearman analysis. Meanwhile, statistical tests were considered significant when *p* < 0.05.

## 3. Results and Discussion

### 3.1. Selection of Extraction Version

Both the buffered and unbuffered versions were employed in previous studies to extract anthelmintics and antibiotics in milk or dairy products [19,25,26,27]. In the current study, the unbuffered version, ammonium version, and buffered version with high (acetate-buffered version) and low (citrate-buffered version) ionic strength were selected for comparison. 

In different versions, acetonitrile was used as the extraction solvent, coupled with different buffer salts, as described in Section 2.2, and the results are shown in Figure S1 in the SM. In the case of antibiotics, the recoveries of the antibiotics were lower under the V1, V2, and V4 systems compared to the V3 system, especially for quinolones, with recoveries around 60% under the V3 system, while those for quinolones were around 20% under the V1, V2, and V4 systems. The recoveries for anthelmintics and MEs for both anthelmintics and antibiotics were similar among the four extraction systems. Therefore, considering the simultaneous extraction of antibiotics and anthelmintics, V3 (citrate-buffered version) was chosen for further optimization.

### 3.2. Extractant Volume

Different acetonitrile volumes (10, 15, and 20 mL) were evaluated based on the recoveries and MEs of the target chemicals, and the results of recoveries and MEs are shown in Figure S2. As shown in Figure S2, the recoveries of the antibiotics and anthelmintics were similar, while those with 15 and 20 mL extractant volumes were slightly better than those for the 10 mL system, especially for quinolone antibiotics. However, with the increase in extractant volume, the MEs for the anthelmintics decreased. Therefore, 20 mL of acetonitrile was selected for simultaneous extraction of both antibiotics and anthelmintics.

### 3.3. Dosage of Dehydrating Agent and NaCl

Anhydrous MgSO_4_ and NaCl are commonly used in the QuEChERS method; anhydrous MgSO_4_ could facilitate the distribution of antibiotics and anthelmintics in the organic phase and stratification of the two phases, while NaCl controls the polarity of the extractants and could increase the selectivity of the extractant [28]. Different amounts of anhydrous MgSO_4_ (1.0, 4.0, 6.0, and 10.0 g) were evaluated in this study, and the results are shown in Figure S3. Obviously, higher antibiotics recoveries were achieved with the addition of 4.0 or 6.0 g anhydrous MgSO_4_, while the recoveries of the anthelmintics and the MEs of all the target chemicals were satisfactory in the case of 4.0 g anhydrous MgSO_4_; therefore, 4.0 g of anhydrous MgSO_4_ was adopted in this study. As shown in Figure S3, the recoveries of the antibiotics and anthelmintics were slightly improved with the addition of NaCl in a 1:4 ratio (*w*:*w*), while the MEs for the target chemicals were satisfactory; therefore, 1.0 g of NaCl was selected in this study.

### 3.4. Buffer Salts Ratio Optimization

For the V3, the additive amount of SCTD was measured by its ratio to SCDS (1 g), including 2:1, 1:1, and 1:2 (*w*:*w*). Generally, the recoveries of the investigated compounds were higher in ratios of 1:2 and 2:1 as compared to that of 1:1. While the 1:2 version achieved better MEs for the antibiotics (Figure S4). Therefore, citrate was added in a ratio of 1:2 (1 g SCDS, *w*/*w*) in this study; meanwhile, the additive amount of SCTD was 2 g.

### 3.5. Purification Process Optimization

A portion of 50.0, 100.0, 150.0, and 200.0 mg of anhydrous MgSO_4_ was used for dehydration again in the purification process, and the results are shown in Figure S5. Satisfactory recoveries and MEs of the anthelmintics were achieved with any additive amount of anhydrous MgSO_4_. However, the recoveries of the antibiotics were higher with the addition of 50 mg anhydrous MgSO_4_, especially for the quinolone and macrolide antibiotics. Therefore, 50.0 mg of anhydrous MgSO_4_ was selected in this study.

Different adsorbents, including PSA, C18, and GCB, were used to evaluate the purification efficiencies, and the results are shown in Figure S6. PSA is effective in removing polar organic acids and some saccharides from non-polar samples; in contrast, C18 is mainly used to extract non-polar and medium-polar compounds from polar samples, for instance, fat, while GCB has a strong affinity for planar structure molecules and can effectively remove some pigments [29].

The recoveries of the quinolone and macrolide antibiotics decreased significantly with the addition of any kind and amount of adsorbents, while the recoveries of the anthelmintics did not show obvious change. In addition, none of the three adsorbents improved the MEs, and the MEs were acceptable (most of the MEs were in the range of 70–120%, while the MEs of ABA, EPR, MOX, ENR, LEVOF, NOR, RFP, SCP, SM2, and OFX ranged from 121% to 140%, and the MEs of BIT, CLO, SDZ, RFP, and DANME were in the range of 141–160%) without further purification. Therefore, only 50.0 mg of anhydrous MgSO_4_ was chosen to complete the purification process.

In a word, the developed QuEChERS method for the simultaneous extraction of the antibiotics and anthelmintics in whole cow milk was as follows: 20 mL of acetonitrile was added to 10.0 g of milk in a 50 mL PP tube, which contained 1 g SCDS, 2 g SCTD, 4 g anhydrous MgSO_4_, and 1 g sodium chloride. After vortexing for 2 min, 1.0 mL of the supernatant was collected into another 1.5 mL PP tube by centrifugation at 4000 r/min for 5 min; then, 50 mg of anhydrous MgSO_4_ was added for purification. After 2 min of vortexing, the extract was centrifuged at 15,000 r/min for 10 min, and 0.5 mL of the supernatant was collected and evaporated to dry under N_2_ and reconstituted to 0.2 mL with methanol-water (1:1, *v*/*v*) for UHPLC-MS/MS analysis. Before extraction, 16 µL of the mixed antibiotic and anthelmintic IS solution at 10 mg L^−1^ were spiked into the whole cow milk sample.

### 3.6. Method Verification

The recoveries, MEs, and precision of the developed method were verified using whole cow milk samples in quintuplicate at a spiking level of 20 µg kg^−1^. The precision of the proposed method was expressed as the percentage relative standard deviation (RSD) of replicate analysis, and the results are given in Table 2. Briefly, the recoveries of the antibiotics were in the range of 79.7–117.2%, with the exception of NOR, which was 53.4%, while those for anthelmintics were in the range of 73.1–105.1%. The MEs for most of the anthelmintics were in the range of 70–120%, except for CLO and BIT, which were 156% for both. However, 8 of 26 selected antibiotics showed higher MEs (121.6–159.4%), while the rest were in the range of 80–120%. Satisfactory RSD was achieved using our developed method, which was in the range of 7.2–18.6% for the antibiotics and anthelmintics. Our developed method generally fits the SANCO guide [30], which suggests that a quantitative method should be demonstrated as being capable of obtaining mean recoveries within the range of 70–120% and RSDs lower than 20% [30]. Although the recovery of NOR is lower than 70%, it is worth noting that NOR has a corresponding internal standard in this study, which could be effectively corrected.

### 3.7. Concentrations of the Antibiotics and Anthelmintics in the Whole Cow Milk Samples

**Figure 1:**
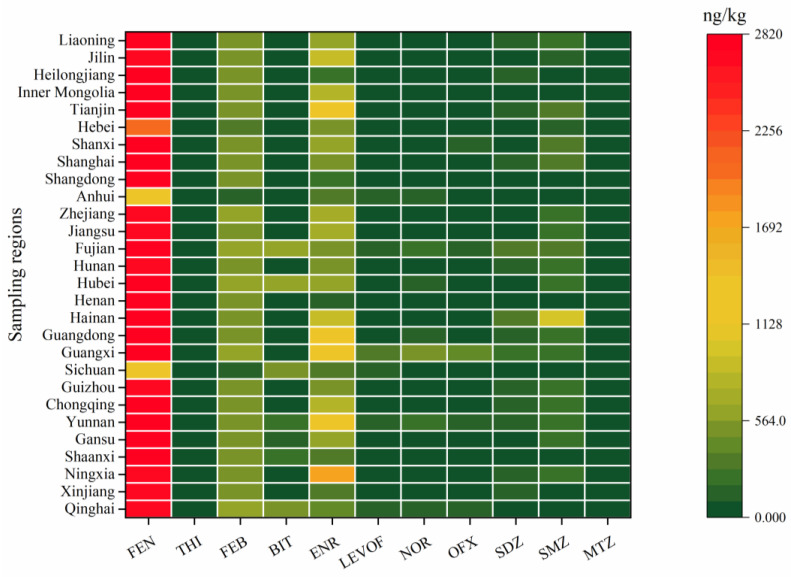
Average concentrations of the antibiotics and anthelmintics (detection frequencies higher than 50%) in the whole cow milk samples from different regions of China.

The concentrations of the investigated anthelmintics in the whole cow milk samples are shown in Figure 1 (only detection frequencies higher than 50% are shown) and Table 3, and the details are given in Table S6. Generally, the detection frequencies and concentrations of the investigated anthelmintics were low in the whole cow milk samples; FLU, MEB, IVE, and MOR were below the LODs in all of the 56 milk samples, while FEN, FEB, and BIT were detected in a frequency of 100%. In addition to THI (98.2%), ALB (30.4%), CLO (33.9%), DOR (21.4%), and DIE (16.1%), the rest of the anthelmintics were found sporadically in the whole cow milk samples with detection frequencies lower than 10%. FEN was the predominant pollutant in the whole cow milk samples, with concentrations ranging from 1159.2 to 2856.5 ng/kg with a median of 2740.5 ng/kg, followed by FEB, at concentrations up to 586.9 and a median of 556.2 ng/kg. Although the detection frequencies were high for BIT and THI, 48 and 54 out of the 56 whole cow milk samples, respectively, contained BIT and THI levels below the LOQs. The remaining anthelmintics also showed a similar phenomenon; although some of them were detected in the milk samples at lower frequencies, most of the concentrations were below the LOQs. A significant positive correlation was found between the concentrations of FEN and FEB; furthermore, FEB could metabolize to FEN in vivo, and FEB is also widely used for the prevention of animal infestations like benzimidazoles [31]. Therefore, relatively higher concentrations of FEN might originate from drug application and metabolism from FEB. No spatial distribution of FEN and BIT levels were found in the whole cow milk samples from different areas of China; however, the whole cow milk samples from North China contained significantly lower FEB than those from Northwest China and Central China, suggesting that anthelmintic application varies in different areas. Most previous studies reported no anthelmintic residues were found in milk samples around the world [19,32,33,34], but FEB was detected in milk samples from Greece with concentrations ranging from 4.9 to 69.7 μg/kg, higher than the results reported in this study [31]. The relatively high concentration of FEB could be attributed to a short time span between the drug administration and the milk withdrawal, since FEB had a short metabolic cycle [35].

A total of 26 antibiotics belonging to six classes were evaluated in the whole cow milk samples from China, and the results are shown in Figure 1 (only detection frequencies higher than 50% are shown) and Table 3, and the details are given in Table S7. Briefly, like anthelmintics, the levels and frequencies of most of the investigated antibiotics were low in the whole cow milk samples. The concentrations of RFP, TYL, and TAP were lower than the LODs in all of the samples. In comparison, ENR and LEVOF were detected in a frequency of 100%, with concentrations ranging from below the LOQs to 4296.8 ng/kg with medians of 513.6 and 53.6 ng/kg, respectively. OFX (98.2%), SDZ (92.9%), and SMZ (92.9%) also had a high detection frequency, with median concentrations of 78.7, 89.7, and 231.5 ng/kg, respectively. NOR, PEN, SCP, DMZ, and MTZ were found in the whole cow milk samples with detection frequencies ranging from 21.4% to 57.1%, and the rest of the antibiotics were sporadically found with detection frequencies lower than 20%. Relatively higher antibiotics detection frequencies were achieved in this study, mainly due to the use of UHPLC-MS/MS analysis methods, which are extremely sensitive compared to HPLC methods used widely in previous studies [36,37]. Generally, quinolones and sulfonamides were the main pollutants, followed by nitroimidazoles; this was consistent with previous studies, which found that sulfonamides and quinolones were the predominant contaminants in Chinese milk [36,38]. It is worth noting that the concentrations of quinolones and sulfonamides were at least one order of magnitude lower than those reported previously in China, Mexico, Spain, and Greece [38,39,40]. Spatial distribution was found for those antibiotics with a detection frequency higher than 50%. Briefly, SDZ levels in the milk samples from South China were significantly higher than those from the remaining areas, with the exception of those from the Northeast, which had similar SDZ concentrations. The milk samples from South China also contained higher levels of ENR, NOR, and SMZ than those from Central, Northeast, and Northwest China. A significantly higher concentration of MTZ was found in the milk samples from the Northwest only as compared to those from East China. In addition, the milk samples from the Southeast had significantly higher concentrations of ENR, LEVOF, NOR, and SMZ than those from the Northwest, the Northeast, North China, and North China, respectively. 

In conclusion, the concentrations of the investigated antibiotics and anthelmintics were all below the Maximum Residue Limits (MRL) suggested by the China Institute of Veterinary Drug Control (GB31650–2019), except for MTZ, which is required to be at no detection. However, based on our analysis method, the LOD and LOQ of the MTZ were substantially lower than those used for the China Institute of Veterinary Drug Control, and the concentration of MTZ in the milk samples was low, with a median of 25.7 ng/kg, which is quite lower than the LOD (μg/L level) of China Institute of Veterinary Drug Control.

### 3.8. Estimated Daily Intake

The EDI values of the investigated antibiotics and anthelmintics via whole cow milk consumption were evaluated based on the current study, and the results are shown in Table 4. Generally, the daily intakes of antibiotics and anthelmintics via whole cow milk consumption for toddlers, teenagers, and adults were far lower than the ADIs (Table S1) suggested by the China Institute of Veterinary Drug Control (GB31650-2019). The EDI values of the antibiotics and anthelmintics via milk consumption for toddlers and children were three times higher than those for adults, suggesting toddlers and children suffer more exposure, mainly due to lower body weight and higher volume of milk intake. However, long-term exposure to low doses of antibiotics and anthelmintics should still be taken seriously, as little is known about its potential effect on the human body, especially for antibiotics, and this needs further study. In addition to milk consumption, numerous anthelmintics and antibiotics were detected in animal-originated food, including pork, chicken, beef, and cultured aquatic products [34,41,42]; therefore, systematic assessment of dietary anthelmintic and antibiotic exposure is the focus of future research.

## 4. Conclusions

An optimized QuEChERS method for the simultaneous extraction of 26 antibiotics and 19 anthelmintics in whole cow milk was successfully established and validated. The extraction was performed using 20 mL acetonitrile with 1 g SCDS, 2 g SCTD, 4 g anhydrous MgSO_4_, and 1 g NaCl. Finally, 50 mg of anhydrous MgSO_4_ was used to purify the extracts. The method was validated and presented high recovery and good precision. The proposed method was successfully applied to the determination of the investigated antibiotics and anthelmintics in 56 whole cow milk samples from China, and the related human exposure to antibiotics and anthelmintics via milk consumption was evaluated. The detection frequencies and concentrations of the investigated anthelmintics and antibiotics were low in the milk samples, with concentrations ranging from below the LOD to 2856.5 ng/kg and from below the LOD to 4296.8 ng/kg, respectively. FEN and FEB were the predominant anthelmintic residues in the milk samples, while quinolones (ENR and LEVOF) and sulfonamides (SDZ and SMZ) were the main antibiotic residues in the milk. The concentrations of the investigated antibiotics and anthelmintics were all below the MRL suggested by the China Institute of Veterinary Drug Control, with the exception of MTZ. Spatial distribution was found for those antibiotics and anthelmintics with detection frequency higher than 50%, indicating regional differences in drug application. Based on the current study, exposure to antibiotics and anthelmintics through whole cow milk consumption are lower than the ADIs suggested by the China Institute of Veterinary Drug Control.

## Figures and Tables

**Table 1:** The parameters of the QuEChERS experiments.

Version	Acetonitrile Volume/mL	Acetic Acid/%	The Ratio of NaOAc to MgSO_4_	The Ratio of NaCl to MgSO_4_	The Ratio of NH_4_Cl to MgSO_4_	The Ratio of SCDS to SCTD
V1	10, 15, 20	0.5, 1, 2, 3	1:2, 1:4, 1:5	/	/	/
V2	10, 15, 20	/	1:2, 1:4, 1:5	/	/	/
V3	10, 15, 20	/	/	1:2, 1:4, 1:5	/	1:1, 2:1, 1:2
V4	10, 15, 20	/	/	/	1:2, 1:4, 1:5	/

**Table 2:** The results of method verification (%).

Anthelmintics	Recovery	Matrix Effect	RSD	Antibiotics	Recovery	Matrix Effect	RSD
ALB	96.7	115.7	8.5	RFP	104.0	159.4	9.5
RIC	93.4	107.4	8.7	ROX	104.0	113.2	14.6
FEN	101.3	112.3	7.8	AZI	106.4	108.8	13.3
OXF	94.6	111.2	8.9	TYL	97.6	99.0	10.6
FLU	94.7	110.1	7.4	PEN	88.1	81.9	15.0
MEB	95.2	112.1	10.5	RNZ	113.1	110.9	10.4
THI	95.2	111.3	7.2	DMZ	116.8	104.2	8.1
ABA	100.6	125.3	10.6	MTZ	106.8	116.1	12.8
DOR	105.1	102.2	10.5	TMP	110.3	105.2	9.4
IVE	99.2	71.7	8.8	SCP	110.3	121.6	9.0
EPR	99.2	151.2	10.1	SDZ	108.7	141.2	13.8
MOX	97.3	130.3	12.2	SMM	111.2	114.3	11.5
LEV	95.6	117.7	12.5	SMR	117.2	99.2	9.1
DIE	73.2	73.4	18.6	SM2	114.0	128.5	12.9
MOR	96.5	71.6	17.5	SMZ	112.1	114.4	15.3
PYR	95.4	113.6	14.0	SPD	104.4	111.0	14.8
FEB	101.4	113.3	13.0	STZ	112.1	107.3	14.0
BIT	80.8	156.0	12.4	ENR	95.4	125.9	16.3
CLO	78.1	156.0	13.6	LEVOF	79.7	133.1	12.2
				DANME	92.2	98.0	7.8
				LOM	79.7	107.0	10.2
				OFX	85.6	133.2	11.0
				NOR	53.4	135.1	11.6
				CHL	110.6	113.0	10.2
				FFC	113.7	115.5	14.3
				TAP	110.3	110.5	11.5

**Table 3:** Median concentration of the anthelmintics and antibiotics in the investigated whole milk samples from different regions of China (ng/kg).

Region	ALB	RIC	FEN	OXF	FLU	MEB	THI	ABA	DOR	IVE	EPR	MOX	LEV	DIE	MOR
Northwest	n.q.	n.d.	2734.6	n.d.	n.d.	n.d.	n.q.	n.d.	n.d.	n.d.	n.d.	n.d.	n.d.	n.d.	n.d.
Southwest	n.d.	n.d.	2750.4	n.d.	n.d.	n.d.	n.q.	n.d.	n.d.	n.d.	n.d.	n.d.	n.d.	n.d.	n.d.
South China	n.q.	n.d.	2752.9	n.d.	n.d.	n.d.	n.q.	n.d.	n.d.	n.d.	n.d.	n.d.	n.d.	n.d.	n.d.
Central China	n.d.	n.d.	2779.9	n.d.	n.d.	n.d.	n.q.	n.d.	n.d.	n.d.	n.d.	n.d.	n.d.	n.d.	n.d.
Eastern China	n.d.	n.d.	2740.4	n.d.	n.d.	n.d.	n.q.	n.d.	n.d.	n.d.	n.d.	n.d.	n.d.	n.d.	n.d.
North China	n.d.	n.d.	2715.1	n.d.	n.d.	n.d.	n.q.	n.d.	n.d.	n.d.	n.d.	n.d.	n.d.	n.d.	n.d.
Northeast	n.d.	n.d.	2736.75	n.d.	n.d.	n.d.	n.q.	n.d.	n.q.	n.d.	n.d.	n.d.	n.d.	n.d.	n.d.
Region	PYR	FEB	BIT	CLO	ENR	LEVOF	NOR	PEN	RFP	ROX	SCP	SDZ	SMM	SMR	SM2
Northwest	n.d.	559.3	n.q.	n.q.	399.5	53.9	17.4	n.d.	n.d.	n.d.	n.d.	89.7	n.d.	n.d.	n.d.
Southwest	n.d.	549.2	n.q.	49.5	784.5	77.5	n.d.	n.d.	n.d.	n.d.	n.d.	78.35	n.d.	n.d.	n.d.
South China	n.d.	562.8	n.q.	n.d.	1378.8	91.6	144.2	n.d.	n.d.	n.d.	186.5	268.1	52.1	n.d.	n.d.
Central China	n.d.	563.4	n.q.	n.d.	221.9	n.q.	n.d.	n.d.	n.d.	n.d.	n.d.	72.4	n.d.	n.d.	n.d.
Eastern China	n.d.	550.6	n.q.	n.d.	428.2	40.15	80.9	n.d.	n.d.	n.d.	n.d.	75.9	n.d.	n.d.	n.d.
North China	n.d.	550.15	n.q.	n.d.	583.5	49.25	n.d.	n.d.	n.d.	n.d.	n.d.	88.5	n.d.	n.d.	n.d.
Northeast	n.d.	548.7	n.q.	n.d.	348.1	35.4	27.7	n.d.	n.d.	n.d.	n.d.	108.2	n.d.	n.d.	n.d.
Region	SMZ	SPD	STZ	TMP	AZI	DANME	DMZ	LOM	MTZ	OFX	RNZ	TYL	CHL	FFC	TAP
Northwest	n.q.	n.d.	n.d.	n.d.	n.d.	n.d.	13.65	n.d.	n.q.	n.q.	n.d.	n.d.	n.d.	n.d.	n.d.
Southwest	212.9	n.d.	n.d.	n.d.	n.d.	n.d.	13.65	n.d.	25.4	n.q.	n.d.	n.d.	n.d.	n.d.	n.d.
South China	252.7	88.3	n.d.	n.d.	n.d.	n.d.	n.d.	n.d.	24	n.q.	n.d.	n.d.	n.d.	n.d.	n.d.
Central China	42.7	n.d.	n.d.	n.d.	n.d.	n.d.	40.8	n.d.	n.d.	n.q.	n.d.	n.d.	n.d.	n.d.	n.d.
Eastern China	122.65	n.d.	n.d.	n.d.	n.d.	n.d.	n.d.	n.d.	n.d.	n.q.	n.d.	n.d.	n.d.	n.d.	n.d.
North China	226.05	n.d.	n.d.	n.d.	n.d.	n.d.	8.875	n.d.	n.d.	n.q.	n.d.	n.d.	n.d.	n.d.	n.d.
Northeast	140.3	n.d.	n.d.	n.d.	n.d.	n.d.	n.d.	n.q.	n.q.	n.q.	n.d.	n.d.	n.d.	n.d.	n.d.

n.d.: below the LOD; n.q.: below the LOQ.

**Table 4:** Daily intakes of antibiotics and anthelmintics via milk consumption (ng/kg/d).

	Toddlers (2–5 Years)		Teenagers (6–17 Years)		Adults (>18 Years)	
	A	B	A	B	A	B
ENR	1.4266	6.6108	0.4016	1.8608	0.1040	0.4818
LEVOF	0.1413	0.6951	0.0398	0.1957	0.0102	0.0506
NOR	0.0388	0.8267	0.0109	0.2327	0.0028	0.0603
OFX	0.0891	0.6743	0.0251	0.1898	0.0065	0.0491
SDZ	0.2462	0.8909	0.0693	0.2508	0.0180	0.0649
SMZ	0.4055	1.2387	0.1141	0.3487	0.0295	0.0903
MTZ	0.0236	0.1312	0.0066	0.0369	0.0017	0.0095
FEN	7.6125	7.8901	2.1428	2.2209	0.5547	0.5750
THI	0.0065	0.0065	0.0018	0.0018	0.0005	0.0005
FEB	1.5449	1.6077	0.4349	0.4525	0.1126	0.1172
BIT	0.0222	2.1831	0.0062	0.6145	0.0016	0.1591

## Data Availability

The data that support the findings of this study are available from the corresponding author upon reasonable request.

## Supplementary Materials

The following supporting information can be downloaded at https://www.mdpi.com/article/10.3390/antibiotics12071125/s1. Figure S1: Recoveries (R) and Matrix effects (ME) of the antibiotics and anthelmintics from different extraction versions; Figure S2: Recoveries (R) and Matrix effects (ME) of the antibiotics and anthelmintics from different volume of extractant; Figure S3: Recoveries (R) and Matrix effects (MEs) of the antibiotics and anthelmintics from different additive amounts of anhydrous MgSO4 for dehydration and different ratio of sodium chloride to anhydrous MgSO4 for buffering.; Figure S4: Recoveries (R) and Matrix effects (MEs) of the antibiotics and anthelmintics from different ratio of SCDS to SCTD for buffering; Figure S5: Recoveries (R) and Matrix effects (MEs) of the antibiotics and anthelmintics from different additive amounts of anhydrous MgSO4 for purification.; Figure S6: Recoveries (R) and Matrix effects (MEs) of the antibiotics and anthelmintics from different additive amounts of GCB, C18 and PSA for purification.; Table S1: Information of the investigated antibiotics and anthelmintics, and their maximum residue limits (MRL) acceptable daily intake (ADI); Table S2: Information of the investigated milk samples; Table S3: Mass spectrometer parameters for determination of antibiotics and anthelmintics; Table S4: The results of QA/QC; Table S5: The parameters for exposure evaluation; Table S6: Concentrations of the anthelmintics in the 56 milk samples from China; Table S7: Concentrations of the antibiotics in the 56 milk samples from China; and Section S1: Details of UHPLC-MS/MS analysis of antibiotics and anthelmintics [43,44,45,46].
