# Supplementary material for: Analysis, Occurrence and Exposure Evaluation of Antibiotic and Anthelmintic Residues in Whole Cow Milk from China
Source: Antibiotics (Basel). 2023 Jun 29;12(7):1125. doi: 10.3390/antibiotics12071125 (PMC10376884; doi:10.3390/antibiotics12071125)
Supplement: Supplementary file 1 [file antibiotics-12-01125-s001.zip › antibiotics-2412928-supplementary.pdf]

# Supplementary Materials

**Section S1** Details of UHPLC-MS/MS analysis of antibiotics and anthelmintics

The electrospray ionization (ESI) interface operated simultaneously in the positive (+) and negative (-) modes. The parameters for the MS system were set as following: curtain gas 35/30 psi (the former parameter was for antibiotics analysis, the latter was for anthelmintics analysis, the same below), ion spray voltage 5500/5000 V, source temperature 500/250 °C and ion source gases (GS1 and GS2) were set to 60/65 psi and 60/35 psi, respectively.

The separation of antibiotics and anthelmintics was achieved using a Acquity UPLC® BEH C18 column (1.7 μm, 2.1×100 mm) with a Acquity UPLC® BEH C18 protection column (1.7 μm, 2.1×5 mm). The mobile phase for antibiotics analysis was 0.1% formic acid in water (A) - methanol (B), and the mobile phase for anthelmintics analysis was 5 mM ammonium formate (containing 0.05% formic acid, A) in water - methanol (B). The column temperature was 35/45°C. The flow rate was 0.3/0.4 mL/min. The mobile phase gradient started with 10% B (methanol), increasing to 100% B at 4.5/3.5 (antibiotics/anthelmintics) min and held for 2.5/3.5 min. Then eluent B was lower back to initial condition over 0.5 min. Finally, the column was equilibrated for 2.5 min. The injection volume was 3/5 μL.

Table S1 Information of the investigated antibiotics and anthelmintics, and their maximum residue limits (MRL) acceptable daily intake (ADI).

| Antibiotics | | CAS NO. | Class | MRL[a] (μg/kg) | ADI[a] (μg/kg BW) | Anthelmintics | | CAS NO. | Class | MRL[a] (μg/kg) | ADI[a] (μg/kg BW) |
|---|---|---|---|---|---|---|---|---|---|---|---|
| Sulfadimethoxine | SMM | 122-11-2 | Sulfonamides | 25 | sum≤50 | Albendazole | ALB | 54965-21-8 | Benzimidazoles | 100 | sum≤50 |
| Sulfachloropyridazine | SCP | 80-32-0 | | sum≤100 | | Ricobendazole | RIC | 54029-12-8 | | - | |
| Sulfathiazole | STZ | 72-14-0 | | | | Flubendazole | FLU | 31430-15-6 | | - | 12 |
| Sulfamethoxazole | SMZ | 723-46-6 | | | | Mebendazole | MEB | 31431-39-7 | | - | 12.5 |
| Sulfamethazine | SM2 | 57-68-1 | | | | Thiabendazole | THI | 148-79-8 | | 100 | 100 |
| Sulfadiazine | SDZ | 68-35-9 | | | | Fenbendazole | FEN | 43210-67-9 | | sum≤100 | sum≤7 |
| Sulfamerazine | SMR | 127-79-7 | | | | Oxfendazole | OXF | 53716-50-0 | | | |
| Sulfapyridine | SPD | 144-83-2 | | | | Febantel | FEB | 58306-30-2 | Diphenylsulfides | | |
| Trimethoprim | TMP | 738-70-5 | | | | Bithionol | BIT | 97-18-7 | | - | - |
| Roxithromycin | ROX | 80214-83-1 | Macrolides | - | - | Levamisole | LEV | 14769-73-4 | Imidazothiazoles | - | 6 |
| Rifampicin | RFP | 13292-46-1 | | - | - | Diethylcarbamazine | DIE | 1642-54-2 | Hexahydropyrazines | - | - |
| Azithromycin dihydrate | AZI | 117772-70-0 | | - | - | Abamectin | ABA | 71751-41-2 | Macrocylic Lactones | - | 2 |
| Tylosin tartrate | TYL | 74610-55-2 | | 100 | 30 | Doramectin | DOR | 117704-25-3 | | 15 | 1 |
| Florfenicol | FFC | 73231-34-2 | Amphenicols | - | 3 | Ivermectin | IVE | 70288-86-7 | | 10 | 10 |
| Thiamphenicol | TAP | 15318-45-3 | | 50 | 5 | Eprinomectin | EPR | 123997-26-2 | | 20 | 10 |
| Chloramphenicol | CHL | 56-75-7 | | - | - | Moxidectin | MOX | 113507-06-5 | | - | 2 |
| Ronidazole | RND | 7681-76-7 | | - | - | Pyrantel | PYR | 15686-83-6 | Tetrahydropyrimidines | - | - |

| | | | | | | | | | | | |
|---|---|---|---|---|---|---|---|---|---|---|---|
| Metronidazole | MTZ | 443-48-1 | Nitroimidazoles | no detection | no detection | Morantel | MOR | 26155-31-7 | | - | - |
| Dimetridazole | DMZ | 551-92-8 | | - | - | Closantel | CLO | 57808-65-8 | Salicylanilides | 45 | 30 |
| Penicillin G potassium salt | PEN | 113-98-4 | β-Lactam | 4 | 30 | | | | | | |
| Enrofloxacin | ENR | 93106-60-6 | Quinolones | 100 | 6.2 | | | | | | |
| Levofloxacin | LEVOF | 100986-85-4 | | | 0.15 | | | | | | |
| Danofloxacin mesylate | DANME | 119478-55-6 | | 30 | 20 | | | | | | |
| Lomefloxacin hydrochloride | LOM | 98079-52-8 | | - | - | | | | | | |
| Ofloxacin | OFX | 82419-36-1 | | - | - | | | | | | |
| Norfloxacin | NOR | 70458-96-7 | | - | 11.4 | | | | | | |

a: data were originated from China Institute of Veterinary Drug Control (GB31650–2019)

Table S2 Information of the investigated whole cow milk samples.

| Region | Province | Code | Sample size |
|---|---|---|---|
| Northwest | Xinjiang | X | 5 |
| | Qinghai | Q | 3 |
| | Gansu | GAN | 3 |
| | Ningxia | N | 3 |
| | Shaanxi | QIN | 3 |
| Southwest | Sichuan | SHU | 1 |
| | Chongqing | YU | 1 |

| | | | |
|---|---|---|---|
| | Yunnan | YUN | 4 |
| | Guizhou | G | 1 |
| | Guangxi | GUI | 1 |
| South China | Guangdong | YUE | 1 |
| | Hainan | QIONG | 1 |
| | Hunan | XIANG | 1 |
| Central China | Hubei | E | 1 |
| | Henan | Y | 3 |
| | Fujian | M | 1 |
| | Zhejiang | Z | 1 |
| Eastern China | Anhui | WAN | 2 |
| | Jiangsu | S | 1 |
| | Shandong | LU | 3 |
| | Shanghai | HU | 2 |
| | Shanxi | JIN | 1 |
| | Hebei | YI | 2 |
| North China | Tianjin | J | 3 |
| | Inner Mongolia | NMG | 2 |
| | Heilongjiang | H | 3 |
| Northeast | Liaoning | L | 2 |
| | Jilin | JI | 1 |

**Table S3** Mass spectrometer parameters for determination of antibiotics and anthelmintics

| Name | ESI mode | Precursor ion (m/z) | Daughter ions (m/z) | DP | EP | CE | CXP | Internal standard |
|---|---|---|---|---|---|---|---|---|

| | | | | | (volts) | (volts) | (volts) | (volts) | |
|---|---|---|---|---|---|---|---|---|---|
| | | | | | Antibiotics | | | | |
| STZ | positive | 256.1 | 156 | 91.9 | 66 | 10 | 21 | 9 | SMZ-D4 |
| SCP | positive | 285 | 155.9 | 91.9 | 64 | 10 | 21 | 9 | SMZ-D4 |
| SMM | positive | 311 | 155.9 | 108.2 | 72 | 10 | 27 | 9 | SMZ-D4 |
| SMZ | positive | 254.1 | 156 | 108 | 67 | 10 | 23 | 9 | SMZ-D4 |
| SMZ-D4 | positive | 258 | 191.9 | | 67 | 10 | 20 | 9 | |
| SM2 | positive | 279.1 | 186.1 | 156 | 72 | 10 | 23 | 9 | SMZ-D4 |
| SDZ | positive | 251 | 156 | 91.8 | 60 | 10 | 21 | 9 | SMZ-D4 |
| SMR | positive | 265.1 | 156 | 172 | 68 | 10 | 23 | 9 | SMZ-D4 |
| SPD | positive | 250.1 | 156 | 184.2 | 66 | 10 | 23 | 9 | SMZ-D4 |
| TMP | positive | 291 | 230.1 | 122.9 | 88 | 10 | 32 | 9 | SMZ-D4 |
| ROX | positive | 837.5 | 679.4 | 558.4 | 88 | 10 | 31 | 9 | ROX-D7 |
| ROX-D7 | positive | 845.6 | 687.6 | | 120 | 10 | 31 | 9 | |
| RFP | positive | 823.4 | 791.4 | 95 | 86 | 10 | 27 | 9 | ROX-D7 |
| AZI | positive | 749.4 | 591.3 | 158 | 156 | 10 | 41 | 9 | ROX-D7 |
| TYL | positive | 916.6 | 174.1 | 772.3 | 126 | 10 | 47 | 9 | ROX-D7 |
| FFC | negative | 356 | 336.1 | 184.6 | -70 | -10 | -14 | -11 | CHL-D5 |
| TAP | negative | 354.1 | 290 | 185.1 | -68 | -10 | -18 | -11 | CHL-D5 |
| CHL | negative | 320.9 | 152.1 | 257.1 | -60 | -10 | -24 | -11 | CHL-D5 |
| CHL-D5 | negative | 326 | 260.8 | | -80 | -10 | -38 | -11 | |
| RND | positive | 201 | 140.3 | 55 | 83 | 10 | 16 | 9 | RND-D3 |
| RND-D3 | positive | 204 | 143 | | 36 | 10 | 17 | 9 | |
| MTZ | positive | 172 | 128.2 | 82 | 60 | 10 | 20 | 9 | RND-D3 |
| DMZ | positive | 142.1 | 96 | 53.9 | 52 | 10 | 21 | 9 | RND-D3 |
| PEN | positive | 335 | 217.3 | 175.8 | 112 | 10 | 20 | 9 | PEN-D5 |

| | | | | | | | | | |
|---|---|---|---|---|---|---|---|---|---|
| PEN-D5 | positive | 340 | 217 | | 118 | 10 | 21 | 9 | |
| ENR | positive | 360 | 316.2 | 245.3 | 90 | 10 | 28 | 9 | NOR-D5 |
| LEVOF | positive | 362.1 | 318 | 261.1 | 92 | 10 | 27 | 9 | NOR-D5 |
| DANME | positive | 358 | 314.1 | 96.1 | 103 | 10 | 25 | 9 | NOR-D5 |
| LOM | positive | 352 | 264.9 | 333.9 | 92 | 10 | 34 | 9 | NOR-D5 |
| OFX | positive | 362 | 318 | 216 | 93 | 10 | 27 | 9 | NOR-D5 |
| NOR | positive | 319.9 | 276.3 | 233.1 | 90 | 10 | 25 | 9 | NOR-D5 |
| NOR-D5 | positive | 325 | 280.9 | | 106 | 10 | 25 | 9 | |
| Anthelmintics | | | | | | | | | |
| ALB | positive | 266 | 234 | 191 | 104 | 10 | 25 | 9 | ALB-D7 |
| ALB-D7 | positive | 273.2 | 241.1 | | 80 | 10 | 29 | 9 | |
| RIC | positive | 282 | 240 | 208 | 52 | 10 | 19 | 9 | ALB-D7 |
| FLU | positive | 314.2 | 282 | 123 | 84 | 10 | 33 | 9 | FEN-D3 |
| MEB | positive | 296.1 | 263.8 | 105.1 | 90 | 10 | 30 | 9 | FEN-D3 |
| THI | positive | 202.1 | 175 | 131.2 | 104.3 | 10 | 38 | 9 | THI-D4 |
| THI-D4 | positive | 206 | 179 | | 95 | 10 | 36 | 9 | |
| FEN | positive | 300 | 268.1 | 158.9 | 94 | 10 | 30 | 9 | FEND3 |
| FEN-D3 | positive | 303.2 | 268 | | 88 | 10 | 30 | 9 | |
| OXF | positive | 316.2 | 158.9 | 191.1 | 77 | 10 | 46 | 9 | FEN-D3 |
| FEB | positive | 447.1 | 383.1 | 415 | 80 | 10 | 26 | 9 | FEB-D6 |
| FEB-D6 | positive | 453.1 | 383.1 | | 88 | 10 | 28 | 9 | |
| BIT | negative | 352.9 | 160.9 | 191.9 | -44 | -10 | -34 | -11 | FEB-D6 |
| LEV | positive | 205.1 | 178 | 123.1 | 88 | 10 | 27 | 9 | LEV-D5 |
| LEV-D5 | positive | 210.1 | 183.1 | | 84 | 10 | 30 | 9 | |
| DIE | positive | 200.1 | 100.2 | 127.1 | 61 | 10 | 22 | 9 | DIE-D3 |
| DIE-D3 | positive | 203 | 100.1 | | 62 | 10 | 22 | 9 | |

| | | | | | | | | | |
|---|---|---|---|---|---|---|---|---|---|
| ABA | positive | 890.6 | 567.3 | 305.2 | 92 | 10 | 20 | 9 | ROX-D7 |
| DOR | positive | 916.5 | 331.2 | 593.3 | 95 | 10 | 34 | 9 | ROX-D7 |
| IVE | positive | 892.5 | 569.3 | 307.1 | 100 | 10 | 22 | 9 | ROX-D7 |
| EPR | positive | 914.5 | 186.2 | 154.1 | 108 | 10 | 25 | 9 | ROX-D7 |
| MOX | positive | 640.4 | 528.2 | 498.3 | 92 | 10 | 14 | 9 | ROX-D7 |
| PYR | positive | 207 | 150 | 136.1 | 84 | 10 | 38 | 9 | LEV-D5 |
| MOR | positive | 221.1 | 122.9 | 164.2 | 84 | 10 | 45 | 9 | LEVD5 |
| CLO | negative | 660.7 | 126.9 | 344.8 | -108 | -10 | -113 | -11 | CLO-C6 |
| CLO-C6 | negative | 666.5 | 126.9 | | -120 | -10 | -104 | -11 | |

Table S4 The results of QA/QC

| Anthelmintics | LOD (ng/kg) | LOQ (ng/kg) | 10 μg/L standard (RSD) | Antibiotics | LOD (ng/kg) | LOQ (ng/kg) | 10 μg/L standard (RSD) |
|---|---|---|---|---|---|---|---|
| ALB | 1.44 | 4.80 | 9.66 (0.11) | RFP | 10.96 | 36.64 | 9.51 (0.13) |
| RIC | 1.44 | 4.80 | 9.69 (0.05) | ROX | 6.08 | 20.16 | 10.93 (0.16) |
| FEN | 0.48 | 1.60 | 9.97 (0.03) | AZI | 4.16 | 13.92 | 10.82 (0.08) |
| OXF | 1.44 | 4.80 | 10.84 (0.09) | TYL | 12.16 | 40.40 | 10.6 (0.1) |
| FLU | 4.80 | 16.00 | 9.87 (0.1) | PEN | 15.60 | 51.92 | 10.17 (0.13) |
| MEB | 1.44 | 4.80 | 10.54 (0.08) | RND | 10.72 | 35.76 | 9.69 (0.1) |
| THI | 1.44 | 4.80 | 10.05 (0.04) | DMZ | 8.24 | 27.36 | 9.63 (0.16) |
| ABA | 0.48 | 1.60 | 10.37 (0.18) | MTZ | 5.12 | 17.12 | 10.95 (0.07) |
| DOR | 1.44 | 4.80 | 10.32 (0.18) | TMP | 5.12 | 17.04 | 9.53 (0.14) |
| IVE | 4.80 | 16.00 | 9.84 (0.18) | SCP | 33.60 | 111.92 | 10.43 (0.08) |
| EPR | 14.40 | 48.00 | 10.31 (0.18) | SDZ | 5.92 | 19.84 | 10.27 (0.14) |
| MOX | 4.80 | 16.00 | 10.61 (0.09) | SMM | 7.12 | 23.68 | 9.93 (0.13) |
| LEV | 1.44 | 4.80 | 10.87 (0.07) | SMR | 35.44 | 118.16 | 9.79 (0.08) |

| | | | | | | | |
|---|---|---|---|---|---|---|---|
| DIE | 14.40 | 48.00 | 10.36 (0.03) | SM2 | 26.48 | 88.24 | 10.14 (0.12) |
| MOR | 4.80 | 16.00 | 10.2 (0.03) | SMZ | 23.92 | 79.84 | 10.71 (0.14) |
| PYR | 14.40 | 48.00 | 9.82 (0.04) | SPD | 9.76 | 32.56 | 9.58 (0.12) |
| FEB | 14.40 | 48.00 | 9.95 (0.05) | STZ | 10.00 | 33.28 | 10.18 (0.12) |
| BIT | 4.80 | 16.00 | 9.91 (0.18) | ENR | 15.28 | 51.04 | 9.57 (0.07) |
| CLO | 1.44 | 4.80 | 9.92 (0.09) | LEVOF | 8.80 | 29.28 | 9.99 (0.08) |
| | | | | DANME | 35.60 | 118.72 | 9.87 (0.1) |
| | | | | LOM | 25.52 | 84.96 | 9.47 (0.14) |
| | | | | OFX | 19.28 | 64.24 | 10.82 (0.05) |
| | | | | NOR | 8.40 | 28.08 | 9.7 (0.06) |
| | | | | CHL | 15.12 | 50.24 | 10.59 (0.07) |
| | | | | FFC | 15.44 | 51.44 | 10.52 (0.08) |
| | | | | TAP | 47.92 | 159.76 | 10.06 (0.15) |

Table S5 The parameters for exposure evaluation

| | Toddlers (2-5 years) | Teenagers (6-17 years) | Adults (>18 years) |
|---|---|---|---|
| Body wight (kg) | 15[a] | 40[b] | 62[c] |
| Milk consumption (mL) | 40.5[d] | 30.4[d] | 12.2[d] |

a: the data were come from MEP (2015b);

b: the data were come from MEP (2015a);

c: the data were come from MEP (2013);

d: the data were come from Zhai (2008).

Table S6 Concentrations of the anthelmintics in the 56 whole cow milk samples from China.

| Region | sample | (ng/kg) | ALB | RIC | FEN | OXF | FLU | MEB | THI | ABA | DOR | IVE | EPR | MOX | LEV | DIE | MOR | PYR | FEB | BIT | CLO |
|---|---|---|---|---|---|---|---|---|---|---|---|---|---|---|---|---|---|---|---|---|---|
| Northwest | Q-1 | mean | n.q. | n.d. | 2842.3 | n.d. | n.d. | n.d. | n.q. | n.d. | n.d. | n.d. | n.d. | n.d. | n.d. | n.d. | n.d. | n.d. | 586.9 | 1108.0 | 220.6 |
| | | SD | \ | \ | 44.7 | \ | \ | \ | \ | \ | \ | \ | \ | \ | \ | \ | \ | \ | 16.7 | 167.8 | 17.4 |
| | Q-2 | mean | n.q. | n.d. | 2632.4 | n.d. | n.d. | n.d. | n.q. | n.d. | n.d. | n.d. | n.d. | n.d. | n.d. | n.d. | n.d. | n.d. | 564.1 | 306.2 | 50.8 |
| | | SD | \ | \ | 16.3 | \ | \ | \ | \ | \ | \ | \ | \ | \ | \ | \ | \ | \ | 9.0 | 58.3 | 1.4 |
| | Q-3 | mean | n.q. | n.q. | 2670.3 | n.d. | n.d. | n.d. | n.q. | n.d. | n.d. | n.d. | n.d. | n.d. | n.d. | n.d. | n.d. | n.d. | 560.3 | n.q. | n.q. |
| | | SD | \ | \ | 117.7 | \ | \ | \ | \ | \ | \ | \ | \ | \ | \ | \ | \ | \ | 0.4 | \ | \ |
| | X-1 | mean | n.q. | n.d. | 2757.1 | n.d. | n.d. | n.d. | n.q. | n.d. | n.q. | n.d. | n.d. | n.d. | n.d. | n.d. | n.d. | n.d. | 578.4 | n.q. | n.q. |
| | | SD | \ | \ | 28.4 | \ | \ | \ | \ | \ | \ | \ | \ | \ | \ | \ | \ | \ | 12.9 | \ | \ |
| | X-2 | mean | n.q. | n.q. | 2705.7 | n.d. | n.d. | n.d. | n.q. | n.d. | n.q. | n.d. | n.d. | n.d. | n.d. | n.d. | n.d. | n.d. | 557.9 | n.q. | n.q. |
| | | SD | \ | \ | 24.8 | \ | \ | \ | \ | \ | \ | \ | \ | \ | \ | \ | \ | \ | 16.9 | \ | \ |
| | X-3 | mean | n.d. | n.d. | 2673.1 | n.d. | n.d. | n.d. | n.q. | n.d. | n.d. | n.d. | n.d. | n.d. | n.d. | n.d. | n.d. | n.d. | 550.8 | n.q. | n.d. |
| | | SD | \ | \ | 35.0 | \ | \ | \ | \ | \ | \ | \ | \ | \ | \ | \ | \ | \ | 8.9 | \ | \ |
| | X-4 | mean | n.d. | n.d. | 2773.3 | n.d. | n.d. | n.d. | n.q. | n.d. | n.q. | n.d. | n.d. | n.d. | n.d. | n.d. | n.d. | n.d. | 559.3 | n.q. | n.d. |
| | | SD | \ | \ | 120.3 | \ | \ | \ | \ | \ | \ | \ | \ | \ | \ | \ | \ | \ | 8.9 | \ | \ |
| | X-5 | mean | n.d. | n.d. | 2614.8 | n.d. | n.d. | n.d. | n.q. | n.d. | n.d. | n.d. | n.d. | n.d. | n.d. | n.d. | n.d. | n.d. | 564.4 | n.q. | n.d. |
| | | SD | \ | \ | 113.5 | \ | \ | \ | \ | \ | \ | \ | \ | \ | \ | \ | \ | \ | 0.4 | \ | \ |
| | N-1 | mean | n.d. | n.d. | 2683.5 | n.q. | n.d. | n.d. | n.q. | n.d. | n.d. | n.d. | n.d. | n.d. | n.d. | n.d. | n.d. | n.d. | 557.8 | n.q. | 48.1 |
| | | SD | \ | \ | 29.7 | \ | \ | \ | \ | \ | \ | \ | \ | \ | \ | \ | \ | \ | 15.6 | \ | 2.0 |
| | N-2 | mean | n.q. | n.d. | 2782.6 | n.q. | n.d. | n.d. | n.q. | n.d. | n.d. | n.d. | n.d. | n.d. | n.d. | 364.7 | n.d. | n.d. | 569.0 | n.q. | n.q. |
| | | SD | \ | \ | 72.6 | \ | \ | \ | \ | \ | \ | \ | \ | \ | \ | 29.9 | \ | \ | 12.0 | \ | \ |
| | N-3 | mean | n.d. | n.d. | 2704.9 | n.d. | n.d. | n.d. | n.q. | n.d. | n.d. | n.d. | n.d. | n.d. | n.d. | n.d. | n.d. | n.d. | 550.8 | n.q. | n.d. |
| | | SD | \ | \ | 100.3 | \ | \ | \ | \ | \ | \ | \ | \ | \ | \ | \ | \ | \ | 25.2 | \ | \ |
| | QIN-1 | mean | n.q. | n.d. | 2800.5 | n.d. | n.d. | n.d. | n.q. | n.d. | n.d. | n.d. | n.d. | n.d. | n.d. | n.q. | n.d. | n.d. | 547.0 | 780.7 | 78.4 |
| | | SD | \ | \ | 49.5 | \ | \ | \ | \ | \ | \ | \ | \ | \ | \ | \ | \ | \ | 6.2 | 76.7 | 9.8 |

| | | | | | | | | | | | | | | | | | | | | | |
|---|---|---|---|---|---|---|---|---|---|---|---|---|---|---|---|---|---|---|---|---|---|
| | QIN-2 | mean | n.q. | n.d. | 2800.2 | n.q. | n.d. | n.d. | n.q. | n.d. | n.d. | n.d. | n.d. | n.d. | n.d. | n.d. | n.d. | n.d. | 565.4 | n.q. | 45.8 |
| | | SD | \ | \ | 115.1 | \ | \ | \ | \ | \ | \ | \ | \ | \ | \ | \ | \ | \ | 3.0 | \ | 0.9 |
| | QIN-3 | mean | n.q. | n.d. | 2632.4 | n.d. | n.d. | n.d. | n.q. | n.d. | n.q. | n.d. | n.d. | n.d. | n.d. | n.d. | n.d. | n.d. | 569.7 | n.q. | n.d. |
| | | SD | \ | \ | 27.1 | \ | \ | \ | \ | \ | \ | \ | \ | \ | \ | \ | \ | \ | 11.3 | \ | \ |
| | GAN-1 | mean | n.d. | n.d. | 2734.6 | n.d. | n.d. | n.d. | n.q. | n.d. | n.d. | n.d. | n.d. | n.d. | n.d. | n.d. | n.d. | n.d. | 544.0 | 307.2 | n.d. |
| | | SD | \ | \ | 56.2 | \ | \ | \ | \ | \ | \ | \ | \ | \ | \ | \ | \ | \ | 12.3 | 51.2 | \ |
| | GAN-2 | mean | n.d. | n.d. | 2828.5 | n.d. | n.d. | n.d. | n.q. | n.d. | 374.0 | n.d. | n.d. | n.d. | n.d. | n.d. | n.d. | n.d. | 544.0 | n.q. | n.d. |
| | | SD | \ | \ | 16.1 | \ | \ | \ | \ | \ | 59.2 | \ | \ | \ | \ | \ | \ | \ | 14.9 | \ | \ |
| | GAN-3 | mean | n.d. | n.d. | 2840.2 | n.d. | n.d. | n.d. | n.q. | n.d. | n.d. | n.d. | n.d. | n.d. | n.d. | n.q. | n.d. | n.d. | 559.0 | n.q. | n.q. |
| | | SD | \ | \ | 33.1 | \ | \ | \ | \ | \ | \ | \ | \ | \ | \ | \ | \ | \ | 4.4 | \ | \ |
| | YUN-1 | mean | n.q. | n.d. | 2856.5 | n.d. | n.d. | n.d. | n.q. | 1271.9 | n.d. | n.d. | n.d. | n.d. | n.d. | n.d. | n.d. | n.d. | 564.0 | 815.3 | 110.7 |
| | | SD | \ | \ | 131.2 | \ | \ | \ | \ | 122.0 | \ | \ | \ | \ | \ | \ | \ | \ | 7.8 | 49.7 | 11.7 |
| | YUN-2 | mean | n.q. | n.d. | 2767.4 | n.d. | n.d. | n.d. | n.q. | n.d. | n.d. | n.d. | n.d. | n.d. | n.q. | n.q. | n.d. | n.d. | 549.2 | n.q. | 55.4 |
| | | SD | \ | \ | 105.8 | \ | \ | \ | \ | \ | \ | \ | \ | \ | \ | \ | \ | \ | 5.0 | \ | 3.0 |
| | YUN-3 | mean | n.d. | 8.8 | 2761.4 | n.d. | n.d. | n.d. | n.q. | n.q. | n.d. | n.d. | n.d. | n.d. | n.d. | n.d. | n.d. | n.d. | 547.2 | n.q. | n.q. |
| | | SD | \ | 1.7 | 19.9 | \ | \ | \ | \ | \ | \ | \ | \ | \ | \ | \ | \ | \ | 6.8 | \ | \ |
| Southwest | YUN-4 | mean | n.d. | n.d. | 2740.9 | n.q. | n.d. | n.d. | n.q. | n.d. | n.d. | n.d. | n.d. | n.d. | 26.6 | n.q. | n.d. | n.d. | 550.2 | n.q. | 49.5 |
| | | SD | \ | \ | 112.1 | \ | \ | \ | \ | \ | \ | \ | \ | \ | 4.7 | \ | \ | \ | 9.2 | \ | 1.9 |
| | YU-1 | mean | n.d. | n.d. | 2750.4 | n.d. | n.d. | n.d. | n.q. | n.d. | 264.0 | n.d. | n.d. | n.d. | 2965.7 | n.d. | n.d. | n.d. | 538.1 | n.q. | n.d. |
| | | SD | \ | \ | 17.5 | \ | \ | \ | \ | \ | 26.2 | \ | \ | \ | 123.5 | \ | \ | \ | 5.6 | \ | \ |
| | G-1 | mean | n.d. | n.d. | 2668.1 | n.d. | n.d. | n.d. | n.q. | n.d. | n.d. | n.d. | n.d. | n.d. | n.d. | n.d. | n.d. | n.d. | 560.6 | n.q. | n.d. |
| | | SD | \ | \ | 66.7 | \ | \ | \ | \ | \ | \ | \ | \ | \ | \ | \ | \ | \ | 4.5 | \ | \ |
| | SHU-1 | mean | n.q. | n.d. | 1236.3 | n.d. | n.d. | n.d. | n.q. | n.d. | n.d. | n.d. | n.d. | n.d. | n.d. | n.d. | n.d. | n.d. | 187.1 | 486.7 | 124.8 |
| | | SD | \ | \ | 164.5 | \ | \ | \ | \ | \ | \ | \ | \ | \ | \ | \ | \ | \ | 1.7 | 26.3 | 20.8 |
| South | GUI-1 | mean | n.d. | n.d. | 2734.7 | n.d. | n.d. | n.d. | n.q. | n.d. | n.d. | n.d. | n.d. | n.d. | n.d. | n.d. | n.d. | n.d. | 567.8 | n.q. | n.d. |
| China | | SD | \ | \ | 38.5 | \ | \ | \ | \ | \ | \ | \ | \ | \ | \ | \ | \ | \ | 25.7 | \ | \ |

| | | | | | | | | | | | | | | | | | | | | | |
|---|---|---|---|---|---|---|---|---|---|---|---|---|---|---|---|---|---|---|---|---|---|
| | YUE-1 | mean | n.q. | n.d. | 2773.9 | n.q. | n.d. | n.d. | n.q. | n.d. | n.d. | n.d. | n.d. | n.d. | n.d. | n.q. | n.d. | 271.2 | 562.8 | n.q. | n.q. |
| | | SD | \ | \ | 52.4 | \ | \ | \ | \ | \ | \ | \ | \ | \ | \ | \ | \ | 16.9 | 0.6 | \ | \ |
| | QIONG-1 | mean | n.q. | n.d. | 2752.9 | n.d. | n.d. | n.d. | n.q. | n.q. | n.d. | n.d. | n.d. | n.d. | n.d. | n.d. | n.d. | n.d. | 554.8 | n.q. | n.d. |
| | | SD | \ | \ | 3.8 | \ | \ | \ | \ | \ | \ | \ | \ | \ | \ | \ | \ | \ | 15.9 | \ | \ |
| | Y-1 | mean | n.d. | n.d. | 2779.9 | n.d. | n.d. | n.d. | n.q. | n.d. | 332.5 | n.d. | n.d. | n.d. | n.d. | n.d. | n.d. | n.d. | 553.1 | n.q. | 51.1 |
| | | SD | \ | \ | 25.8 | \ | \ | \ | \ | \ | 12.5 | \ | \ | \ | \ | \ | \ | \ | 16.3 | \ | 2.1 |
| | Y-2 | mean | n.d. | n.d. | 2786.4 | n.d. | n.d. | n.d. | n.q. | n.d. | n.d. | n.d. | n.d. | n.d. | n.d. | n.d. | n.d. | n.d. | 564.7 | n.q. | n.d. |
| | | SD | \ | \ | 45.9 | \ | \ | \ | \ | \ | \ | \ | \ | \ | \ | \ | \ | \ | 13.2 | \ | \ |
| Central | Y-3 | mean | n.d. | n.d. | 2655.7 | n.d. | n.d. | n.d. | n.q. | n.d. | n.d. | n.d. | n.d. | n.d. | n.d. | n.d. | n.d. | n.d. | 563.4 | n.q. | n.d. |
| China | | SD | \ | \ | 18.8 | \ | \ | \ | \ | \ | \ | \ | \ | \ | \ | \ | \ | \ | 1.8 | \ | \ |
| | E-1 | mean | n.d. | n.d. | 2787.5 | n.d. | n.d. | n.d. | n.d. | n.d. | n.d. | n.d. | n.d. | n.d. | n.d. | n.d. | n.d. | n.d. | 580.7 | 641.6 | n.q. |
| | | SD | \ | \ | 56.3 | \ | \ | \ | \ | \ | \ | \ | \ | \ | \ | \ | \ | \ | 3.1 | 41.6 | \ |
| | XIANG-1 | mean | n.d. | n.d. | 2680.3 | n.d. | n.d. | n.d. | n.q. | n.d. | n.d. | n.d. | n.d. | n.d. | n.d. | n.d. | n.d. | n.d. | 556.5 | n.q. | n.d. |
| | | SD | \ | \ | 23.9 | \ | \ | \ | \ | \ | \ | \ | \ | \ | \ | \ | \ | \ | 6.2 | \ | \ |
| | M-1 | mean | n.q. | n.d. | 2810.6 | n.d. | n.d. | n.d. | 22.8 | n.d. | n.d. | n.d. | n.d. | n.q. | n.q. | n.q. | n.d. | 277.7 | 569.2 | 569.3 | 77.8 |
| | | SD | \ | \ | 56.8 | \ | \ | \ | 5.1 | \ | \ | \ | \ | \ | \ | \ | \ | 17.1 | 5.7 | 123.1 | 11.7 |
| | S-1 | mean | n.d. | n.d. | 2715.2 | n.d. | n.d. | n.d. | n.q. | n.d. | n.d. | n.d. | n.d. | n.d. | n.d. | n.d. | n.d. | n.d. | 549.3 | n.q. | n.d. |
| | | SD | \ | \ | 12.4 | \ | \ | \ | \ | \ | \ | \ | \ | \ | \ | \ | \ | \ | 2.7 | \ | \ |
| | Z-1 | mean | n.d. | n.d. | 2698.2 | n.d. | n.d. | n.d. | n.q. | n.d. | n.d. | n.d. | n.d. | n.d. | n.d. | n.d. | n.d. | n.d. | 565.1 | n.q. | n.d. |
| Eastern | | SD | \ | \ | 116.2 | \ | \ | \ | \ | \ | \ | \ | \ | \ | \ | \ | \ | \ | 1.0 | \ | \ |
| China | WAN-1 | mean | n.d. | n.d. | 1159.2 | n.d. | n.d. | n.d. | n.q. | n.d. | n.d. | n.d. | n.d. | n.d. | n.d. | n.d. | n.d. | n.d. | 182.3 | n.q. | n.d. |
| | | SD | \ | \ | 168.6 | \ | \ | \ | \ | \ | \ | \ | \ | \ | \ | \ | \ | \ | 1.6 | \ | \ |
| | WAN-2 | mean | n.d. | n.d. | 1355.6 | n.d. | n.d. | n.d. | n.q. | n.d. | n.d. | n.d. | n.d. | n.d. | n.d. | n.d. | n.d. | n.d. | 181.6 | n.q. | n.d. |
| | | SD | \ | \ | 25.4 | \ | \ | \ | \ | \ | \ | \ | \ | \ | \ | \ | \ | \ | 2.2 | \ | \ |
| | LU-1 | mean | n.d. | n.d. | 2764.5 | n.d. | n.d. | n.d. | n.q. | n.d. | n.d. | n.d. | n.d. | n.d. | n.d. | n.d. | n.d. | n.d. | 556.4 | n.q. | n.d. |
| | | SD | \ | \ | 115.8 | \ | \ | \ | \ | \ | \ | \ | \ | \ | \ | \ | \ | \ | 1.5 | \ | \ |

| | | | | | | | | | | | | | | | | | | | | | |
|---|---|---|---|---|---|---|---|---|---|---|---|---|---|---|---|---|---|---|---|---|---|
| | LU-2 | mean | n.d. | n.d. | 2787.1 | n.d. | n.d. | n.d. | n.q. | n.d. | n.d. | n.d. | n.d. | n.d. | n.d. | n.q. | n.d. | n.d. | 573.4 | n.q. | n.d. |
| | | SD | \ | \ | 35.9 | \ | \ | \ | \ | \ | \ | \ | \ | \ | \ | \ | \ | \ | 13.1 | \ | \ |
| | LU-3 | mean | n.q. | n.d. | 2783.5 | n.d. | n.d. | n.d. | n.q. | n.d. | n.d. | n.d. | n.d. | n.d. | n.d. | n.d. | n.d. | n.d. | 541.6 | n.q. | n.d. |
| | | SD | \ | \ | 77.5 | \ | \ | \ | \ | \ | \ | \ | \ | \ | \ | \ | \ | \ | 3.4 | \ | \ |
| | HU-1 | mean | n.d. | n.d. | 2745.2 | n.d. | n.d. | n.d. | n.q. | n.d. | n.d. | n.d. | n.d. | n.d. | n.d. | n.d. | n.d. | n.d. | 532.9 | n.q. | n.d. |
| | | SD | \ | \ | 34.1 | \ | \ | \ | \ | \ | \ | \ | \ | \ | \ | \ | \ | \ | 9.6 | \ | \ |
| | HU-2 | mean | n.d. | n.d. | 2735.6 | n.d. | n.d. | n.d. | n.q. | n.d. | n.d. | n.d. | n.d. | n.d. | n.d. | n.d. | n.d. | n.d. | 551.9 | n.q. | n.d. |
| | | SD | \ | \ | 4.9 | \ | \ | \ | \ | \ | \ | \ | \ | \ | \ | \ | \ | \ | 25.5 | \ | \ |
| North China | JIN-1 | mean | n.d. | n.d. | 2789.1 | n.d. | n.d. | n.d. | n.q. | n.d. | n.d. | n.d. | n.d. | n.d. | n.d. | n.q. | n.d. | n.d. | 548.3 | n.q. | n.d. |
| | | SD | \ | \ | 42.9 | \ | \ | \ | \ | \ | \ | \ | \ | \ | \ | \ | \ | \ | 12.2 | \ | \ |
| | YI-1 | mean | n.d. | n.d. | 2707.3 | n.d. | n.d. | n.d. | n.q. | n.d. | n.d. | n.d. | 246.0 | n.d. | n.d. | n.d. | n.d. | n.d. | 556.0 | n.q. | n.d. |
| | | SD | \ | \ | 15.5 | \ | \ | \ | \ | \ | \ | \ | 8.7 | \ | \ | \ | \ | \ | 3.2 | \ | \ |
| | YI-2 | mean | n.d. | n.d. | 1350.7 | n.d. | n.d. | n.d. | n.q. | n.d. | n.d. | n.d. | n.d. | n.d. | n.d. | n.d. | n.d. | n.d. | 183.6 | n.q. | n.d. |
| | | SD | \ | \ | 17.6 | \ | \ | \ | \ | \ | \ | \ | \ | \ | \ | \ | \ | \ | 3.5 | \ | \ |
| | J-1 | mean | n.d. | n.d. | 2685.4 | n.d. | n.d. | n.d. | n.q. | n.d. | n.d. | n.d. | n.d. | n.d. | n.d. | n.d. | n.d. | n.d. | 549.9 | n.q. | n.d. |
| | | SD | \ | \ | 23.2 | \ | \ | \ | \ | \ | \ | \ | \ | \ | \ | \ | \ | \ | 2.9 | \ | \ |
| | J-2 | mean | n.d. | n.d. | 2803.6 | n.d. | n.d. | n.d. | n.q. | n.d. | 365.7 | n.d. | n.d. | n.d. | n.d. | n.d. | n.d. | n.d. | 558.0 | n.q. | n.d. |
| | | SD | \ | \ | 116.3 | \ | \ | \ | \ | \ | 5.7 | \ | \ | \ | \ | \ | \ | \ | 8.1 | \ | \ |
| | J-3 | mean | n.d. | n.d. | 2689.2 | n.d. | n.d. | n.d. | n.q. | n.d. | n.d. | n.d. | n.d. | n.d. | n.d. | n.d. | n.d. | n.d. | 550.4 | n.q. | n.d. |
| | | SD | \ | \ | 60.8 | \ | \ | \ | \ | \ | \ | \ | \ | \ | \ | \ | \ | \ | 16.0 | \ | \ |
| | NMG-1 | mean | n.d. | n.d. | 2722.9 | n.d. | n.d. | n.d. | n.q. | n.d. | n.d. | n.d. | n.d. | n.d. | n.d. | n.d. | n.d. | n.d. | 546.0 | n.q. | n.d. |
| | | SD | \ | \ | 104.5 | \ | \ | \ | \ | \ | \ | \ | \ | \ | \ | \ | \ | \ | 11.0 | \ | \ |
| | NMG-2 | mean | n.d. | n.d. | 2792.8 | n.d. | n.d. | n.d. | n.q. | n.d. | 302.4 | n.d. | n.d. | n.d. | n.d. | n.d. | n.d. | n.d. | 559.4 | n.q. | n.d. |
| | | SD | \ | \ | 111.8 | \ | \ | \ | \ | \ | 11.5 | \ | \ | \ | \ | \ | \ | \ | 26.0 | \ | \ |
| Northeast | H-1 | mean | n.d. | n.d. | 2729.6 | n.d. | n.d. | n.d. | n.q. | n.d. | n.q. | n.d. | n.d. | n.d. | n.d. | n.d. | n.d. | n.d. | 563.7 | n.q. | n.d. |
| | | SD | \ | \ | 3.3 | \ | \ | \ | \ | \ | \ | \ | \ | \ | \ | \ | \ | \ | 6.0 | \ | \ |

| | | | | | | | | | | | | | | | | | | | | | |
|---|---|---|---|---|---|---|---|---|---|---|---|---|---|---|---|---|---|---|---|---|---|
| | H-2 | mean | n.d. | n.d. | 2733.5 | n.d. | n.d. | n.d. | n.q. | n.d. | n.q. | n.d. | n.d. | n.d. | n.d. | n.d. | n.d. | n.d. | 550.0 | n.q. | n.d. |
| | | SD | \ | \ | 18.5 | \ | \ | \ | \ | \ | \ | \ | \ | \ | \ | \ | \ | \ | 11.0 | \ | \ |
| | H-3 | mean | n.d. | n.d. | 2780.4 | n.d. | n.d. | n.d. | n.q. | n.d. | n.d. | n.d. | n.d. | n.d. | n.d. | n.d. | n.d. | n.d. | 540.7 | n.q. | n.d. |
| | | SD | \ | \ | 111.7 | \ | \ | \ | \ | \ | \ | \ | \ | \ | \ | \ | \ | \ | 5.7 | \ | \ |
| | JI-1 | mean | n.q. | n.d. | 2725.6 | n.d. | n.d. | n.d. | n.q. | n.d. | 327.2 | n.d. | n.d. | n.d. | n.d. | n.d. | n.d. | n.d. | 545.0 | n.q. | n.d. |
| | | SD | \ | \ | 88.2 | \ | \ | \ | \ | \ | 12.4 | \ | \ | \ | \ | \ | \ | \ | 5.0 | \ | \ |
| | L-1 | mean | n.d. | n.d. | 2740.0 | n.d. | n.d. | n.d. | n.q. | n.d. | n.d. | n.d. | n.d. | n.d. | n.d. | n.d. | n.d. | n.d. | 547.4 | n.q. | n.d. |
| | | SD | \ | \ | 27.9 | \ | \ | \ | \ | \ | \ | \ | \ | \ | \ | \ | \ | \ | 5.7 | \ | \ |
| | L-2 | mean | n.d. | n.d. | 2742.0 | n.d. | n.d. | n.d. | n.q. | n.d. | n.d. | n.d. | n.d. | n.d. | n.d. | n.d. | n.d. | n.d. | 559.0 | n.q. | n.d. |
| | | SD | \ | \ | 6.9 | \ | \ | \ | \ | \ | \ | \ | \ | \ | \ | \ | \ | \ | 7.4 | \ | \ |

n.d.: below the LOD; n.q.: below the LOQ.

Table S7 Concentrations of the antibiotics in the 56 whole cow milk samples from China.

| Region | Sample | ng/kg | ENR | LEVOF | NOR | PEN | RFP | ROX | SCP | SDZ | SMM | SMR | SM2 | SMZ | SPD |
|---|---|---|---|---|---|---|---|---|---|---|---|---|---|---|---|
| Northwest | Q-1 | mean | 450.9 | 230.6 | 125.3 | 99.8 | n.d. | n.d. | n.q. | 24.6 | n.d. | n.d. | n.d. | n.q. | n.d. |
| | | SD | 47.8 | 0.9 | 18.4 | 6.0 | \ | \ | \ | 0.1 | \ | \ | \ | \ | \ |
| | Q-2 | mean | 286.6 | 62.9 | n.q. | n.d. | n.d. | n.d. | n.d. | 23.5 | n.d. | 54.8 | 59.5 | 33.8 | n.d. |
| | | SD | 13.6 | 5.7 | \ | \ | \ | \ | \ | 0.8 | \ | 2.8 | 8.9 | 3.0 | \ |
| | Q-3 | mean | 399.5 | 53.9 | 143.1 | n.d. | n.d. | n.d. | n.q. | 38.5 | n.d. | n.d. | n.d. | n.q. | n.d. |
| | | SD | 49.9 | 2.5 | 13.4 | \ | \ | \ | \ | 2.7 | \ | \ | \ | \ | \ |
| | X-1 | mean | 301.8 | 47.8 | 110.5 | n.d. | n.d. | n.d. | n.d. | 107.5 | n.d. | n.d. | n.d. | 77.1 | n.d. |
| | | SD | 52.1 | 1.0 | 73.0 | \ | \ | \ | \ | 22.0 | \ | \ | \ | 9.8 | \ |
| | X-2 | mean | 811.7 | 86.6 | 42.2 | 82.5 | n.d. | n.d. | n.d. | 180.8 | n.d. | n.d. | n.d. | 75.7 | n.d. |
| | | SD | 65.8 | 11.6 | 6.3 | 5.6 | \ | \ | \ | 17.0 | \ | \ | \ | 2.8 | \ |
| | X-3 | mean | 205.6 | 50.3 | n.d. | n.d. | n.d. | n.d. | n.d. | 69.5 | 27.1 | n.d. | n.d. | 39.3 | n.d. |
| | | SD | 5.5 | 7.8 | \ | \ | \ | \ | \ | 2.7 | 0.8 | \ | \ | 5.4 | \ |

| | | | | | | | | | | | | | | |
|---|---|---|---|---|---|---|---|---|---|---|---|---|---|---|
| | X-4 | mean | 245.8 | 63.3 | n.d. | n.d. | n.d. | n.d. | n.d. | 72.3 | n.d. | n.d. | n.d. | n.q. | n.d. |
| | | SD | 8.8 | 7.4 | \ | \ | \ | \ | \ | 13.2 | \ | \ | \ | \ | \ |
| | X-5 | mean | 238.6 | 14.1 | n.q. | n.d. | n.d. | n.d. | n.d. | 140.6 | n.d. | n.d. | n.d. | 69.9 | n.d. |
| | | SD | 10.4 | 1.4 | \ | \ | \ | \ | \ | 1.3 | \ | \ | \ | 8.9 | \ |
| | N-1 | mean | 590.7 | 121.9 | n.d. | 80.8 | n.d. | n.d. | n.d. | 90.1 | n.d. | n.d. | n.d. | n.q. | n.d. |
| | | SD | 51.0 | 16.3 | \ | 8.9 | \ | \ | \ | 1.7 | \ | \ | \ | \ | \ |
| | N-2 | mean | 4296.8 | 91.7 | n.d. | n.d. | n.d. | n.d. | n.d. | 284.6 | n.d. | n.d. | n.d. | 504.4 | n.d. |
| | | SD | 646.3 | 4.7 | \ | \ | \ | \ | \ | 16.6 | \ | \ | \ | 71.2 | \ |
| | N-3 | mean | 466.2 | 31.1 | n.q. | 71.8 | n.d. | n.d. | n.d. | 89.7 | n.d. | n.d. | n.d. | n.q. | n.d. |
| | | SD | 11.9 | 4.1 | \ | 3.6 | \ | \ | \ | 3.9 | \ | \ | \ | \ | \ |
| | QIN-1 | mean | 577.0 | 124.2 | 17.4 | 109.9 | n.d. | n.d. | n.d. | 119.2 | n.d. | n.d. | n.d. | n.q. | n.q. |
| | | SD | 7.6 | 1.5 | 0.4 | 10.4 | \ | \ | \ | 16.1 | \ | \ | \ | \ | \ |
| | QIN-2 | mean | 208.8 | 53.3 | 43.4 | n.d. | n.d. | n.d. | n.d. | 52.9 | n.d. | n.d. | n.d. | n.q. | n.d. |
| | | SD | 11.2 | 6.3 | 6.0 | \ | \ | \ | \ | 3.5 | \ | \ | \ | \ | \ |
| | QIN-3 | mean | 239.8 | 51.3 | 18.0 | n.d. | n.d. | n.d. | n.d. | 105.9 | n.d. | n.d. | n.d. | 56.6 | n.d. |
| | | SD | 15.2 | 8.7 | 0.3 | \ | \ | \ | \ | 5.0 | \ | \ | \ | 9.6 | \ |
| | GAN-1 | mean | 391.0 | 38.3 | n.d. | n.d. | n.d. | n.d. | n.d. | 90.7 | n.d. | n.d. | n.d. | 280.5 | n.d. |
| | | SD | 30.0 | 1.9 | \ | \ | \ | \ | \ | 2.9 | \ | \ | \ | 26.7 | \ |
| | GAN-2 | mean | 655.7 | 32.1 | 93.2 | 439.2 | n.d. | n.d. | n.d. | 88.8 | n.d. | n.d. | 76.3 | 215.2 | n.d. |
| | | SD | 74.8 | 5.6 | 4.8 | 57.7 | \ | \ | \ | 0.9 | \ | \ | 4.5 | 13.3 | \ |
| | GAN-3 | mean | 679.6 | 136.0 | 114.0 | 375.2 | n.d. | n.d. | n.d. | 70.2 | n.d. | n.d. | n.d. | 206.9 | n.d. |
| | | SD | 66.9 | 17.7 | 6.4 | 6.3 | \ | \ | \ | 6.2 | \ | \ | \ | 29.6 | \ |
| Southwest | YUN-1 | mean | 2318.4 | 463.9 | 595.6 | n.d. | n.d. | n.d. | n.d. | 35.5 | 32.8 | n.d. | n.d. | 212.9 | n.d. |
| | | SD | 2.8 | 18.6 | 89.3 | \ | \ | \ | \ | 3.2 | 1.2 | \ | \ | 36.1 | \ |
| | YUN-2 | mean | 1048.9 | 114.9 | 262.4 | n.d. | n.d. | n.d. | n.d. | 32.5 | n.d. | n.d. | n.d. | 200.8 | n.d. |
| | | SD | 24.9 | 3.3 | 40.7 | \ | \ | \ | \ | 1.7 | \ | \ | \ | 10.1 | \ |

| | | | | | | | | | | | | | | | |
|---|---|---|---|---|---|---|---|---|---|---|---|---|---|---|---|
| | YUN-3 | mean | 631.2 | 77.5 | 35.2 | n.d. | n.d. | n.d. | n.d. | 59.4 | n.d. | n.d. | n.d. | 198.1 | n.d. |
| | | SD | 21.4 | 4.7 | 5.5 | \ | \ | \ | \ | 0.3 | \ | \ | \ | 1.7 | \ |
| | YUN-4 | mean | 866.2 | 67.0 | n.d. | 403.9 | n.d. | n.d. | n.d. | 455.9 | n.d. | n.d. | 70.2 | 378.6 | n.d. |
| | | SD | 98.6 | 13.9 | \ | 40.7 | \ | \ | \ | 29.3 | \ | \ | 4.0 | 55.3 | \ |
| | YU-1 | mean | 784.5 | 48.8 | n.d. | n.d. | n.d. | n.d. | n.d. | 97.3 | n.d. | n.d. | n.d. | 238.2 | n.d. |
| | | SD | 71.2 | 7.7 | \ | \ | \ | \ | \ | 11.5 | \ | \ | \ | 3.8 | \ |
| | SHU-1 | mean | 297.1 | 125.0 | n.d. | n.d. | n.d. | n.d. | n.d. | n.d. | n.d. | n.d. | n.d. | n.d. | n.d. |
| | | SD | 4.2 | 20.1 | \ | \ | \ | \ | \ | \ | \ | \ | \ | \ | \ |
| | G-1 | mean | 513.5 | 37.4 | n.d. | n.d. | n.d. | n.d. | n.d. | 108.5 | n.d. | n.d. | n.d. | 235.1 | n.d. |
| | | SD | 70.9 | 6.5 | \ | \ | \ | \ | \ | 1.7 | \ | \ | \ | 26.5 | \ |
| South China | YUE-1 | mean | 1389.0 | 361.8 | 496.6 | n.d. | n.d. | 80.7 | 186.5 | 268.1 | 52.1 | n.d. | 96.1 | 252.7 | n.d. |
| | | SD | 243.5 | 55.1 | 76.9 | \ | \ | 4.6 | 1.6 | 30.8 | 4.3 | \ | 7.5 | 15.3 | \ |
| | GUI-1 | mean | 1378.8 | 49.2 | 144.2 | n.d. | n.d. | n.d. | n.d. | 127.2 | n.d. | n.d. | n.d. | 245.4 | 88.3 |
| | | SD | 116.9 | 6.9 | 21.9 | \ | \ | \ | \ | 11.4 | \ | \ | \ | 5.3 | 1.8 |
| | QIONG-1 | mean | 878.7 | 91.6 | n.d. | n.d. | n.d. | n.d. | 611.5 | 315.9 | 518.7 | n.d. | n.d. | 975.7 | 175.3 |
| | | SD | 66.8 | 11.5 | \ | \ | \ | \ | 81.9 | 33.1 | 5.1 | \ | \ | 36.6 | 6.3 |
| Central China | Y-1 | mean | 221.9 | 143.3 | n.d. | n.d. | n.d. | n.d. | n.d. | 70.6 | 36.2 | n.d. | n.d. | n.q. | n.d. |
| | | SD | 11.0 | 6.2 | \ | \ | \ | \ | \ | 3.8 | 2.3 | \ | \ | \ | \ |
| | Y-2 | mean | 73.7 | 14.4 | n.d. | n.d. | n.d. | n.d. | n.d. | 54.8 | n.d. | n.d. | n.d. | n.q. | n.q. |
| | | SD | 5.2 | 1.9 | \ | \ | \ | \ | \ | 5.3 | \ | \ | \ | \ | \ |
| | Y-3 | mean | 119.9 | n.q. | n.d. | n.d. | n.d. | n.d. | n.q. | 72.4 | n.d. | n.d. | n.d. | 42.7 | n.d. |
| | | SD | 15.6 | \ | \ | \ | \ | \ | \ | 12.1 | \ | \ | \ | 4.6 | \ |
| | E-1 | mean | 639.0 | 64.2 | 132.0 | n.d. | n.d. | n.d. | n.d. | 89.8 | n.d. | n.d. | n.d. | 266.3 | n.d. |
| | | SD | 10.3 | 2.7 | 10.5 | \ | \ | \ | \ | 14.2 | \ | \ | \ | 15.3 | \ |
| | XIANG-1 | mean | 513.6 | 16.9 | n.d. | n.d. | n.d. | n.d. | n.d. | 100.3 | n.d. | n.d. | n.d. | 229.4 | n.d. |
| | | SD | 57.3 | 1.8 | \ | \ | \ | \ | \ | 12.7 | \ | \ | \ | 5.7 | \ |

| | | | | | | | | | | | | | | | |
|---|---|---|---|---|---|---|---|---|---|---|---|---|---|---|---|
| Eastern China | M-1 | mean | 554.3 | 166.8 | 259.3 | 407.5 | n.d. | 58.2 | 490.3 | 308.0 | 190.8 | 287.7 | n.d. | 371.0 | 512.2 |
| | | SD | 24.0 | 17.0 | 30.0 | 47.2 | \ | 7.4 | 46.9 | 4.8 | 32.4 | 39.8 | \ | 46.8 | 44.3 |
| | Z-1 | mean | 659.0 | 34.6 | 83.1 | n.d. | n.d. | n.d. | n.d. | 59.4 | n.d. | n.d. | n.d. | 205.4 | n.d. |
| | | SD | 50.2 | 2.5 | 0.7 | \ | \ | \ | \ | 5.7 | \ | \ | \ | 24.5 | \ |
| | WAN-1 | mean | 283.2 | 187.5 | 222.5 | n.d. | n.d. | n.d. | n.d. | n.d. | n.d. | n.d. | n.d. | n.d. | n.d. |
| | | SD | 25.8 | 11.5 | 13.4 | \ | \ | \ | \ | \ | \ | \ | \ | \ | \ |
| | WAN-2 | mean | 348.1 | 112.1 | n.d. | n.d. | n.d. | n.d. | n.d. | n.d. | n.d. | n.d. | n.d. | n.d. | n.d. |
| | | SD | 42.5 | 3.6 | \ | \ | \ | \ | \ | \ | \ | \ | \ | \ | \ |
| | S-1 | mean | 684.1 | 55.0 | 86.4 | n.d. | n.d. | n.d. | n.d. | 55.6 | n.d. | n.d. | n.d. | 234.2 | n.d. |
| | | SD | 97.3 | 7.9 | 14.8 | \ | \ | \ | \ | 5.6 | \ | \ | \ | 5.2 | \ |
| | HU-1 | mean | 526.6 | 40.2 | 78.7 | n.d. | n.d. | n.d. | 193.0 | 124.2 | n.d. | n.d. | n.d. | 299.3 | n.d. |
| | | SD | 74.9 | 4.8 | 5.5 | \ | \ | \ | 30.8 | 11.0 | \ | \ | \ | 6.3 | \ |
| | HU-2 | mean | 508.3 | 40.1 | 97.6 | n.d. | n.d. | n.d. | 162.5 | 95.4 | n.d. | n.d. | n.d. | 307.2 | n.d. |
| | | SD | 55.9 | 2.7 | 10.9 | \ | \ | \ | 8.1 | 12.4 | \ | \ | \ | 9.2 | \ |
| | LU-1 | mean | 160.0 | n.q. | n.q. | n.d. | n.d. | n.d. | n.q. | 23.9 | n.d. | n.d. | n.d. | n.q. | n.d. |
| | | SD | 26.6 | \ | \ | \ | \ | \ | \ | 2.3 | \ | \ | \ | \ | \ |
| | LU-2 | mean | 270.0 | 31.1 | n.d. | n.d. | n.d. | n.d. | n.d. | 92.4 | n.d. | n.d. | n.d. | n.q. | n.d. |
| | | SD | 14.5 | 4.0 | \ | \ | \ | \ | \ | 3.4 | \ | \ | \ | \ | \ |
| | LU-3 | mean | 346.5 | 26.9 | 69.1 | n.d. | n.d. | n.d. | n.d. | 24.6 | n.d. | n.d. | n.d. | n.q. | n.d. |
| | | SD | 9.6 | 3.1 | 11.8 | \ | \ | \ | \ | 3.3 | \ | \ | \ | \ | \ |
| North China | NMG-1 | mean | 1319.8 | 13.9 | n.d. | n.d. | n.d. | n.d. | n.d. | 82.4 | n.d. | n.d. | n.d. | 138.3 | n.d. |
| | | SD | 58.0 | 1.7 | \ | \ | \ | \ | \ | 1.2 | \ | \ | \ | 4.6 | \ |
| | NMG-2 | mean | 246.3 | 31.7 | 23.5 | n.d. | n.d. | n.d. | n.d. | 65.7 | n.d. | n.d. | n.d. | 35.9 | n.d. |
| | | SD | 13.4 | 1.6 | 1.2 | \ | \ | \ | \ | 6.7 | \ | \ | \ | 2.5 | \ |
| | JIN-1 | mean | 598.2 | 93.2 | n.d. | n.d. | n.d. | n.d. | 158.6 | 88.5 | n.d. | n.d. | n.d. | 312.7 | n.d. |
| | | SD | 42.5 | 13.6 | \ | \ | \ | \ | 13.6 | 10.0 | \ | \ | \ | 28.1 | \ |

| | | | | | | | | | | | | | | | |
|---|---|---|---|---|---|---|---|---|---|---|---|---|---|---|---|
| | YI-1 | mean | 612.0 | 49.9 | n.d. | 389.0 | n.d. | n.d. | n.d. | 105.9 | n.d. | n.d. | n.d. | 245.3 | n.d. |
| | | SD | 37.8 | 9.4 | \ | 10.9 | \ | \ | \ | 8.3 | \ | \ | \ | 43.0 | \ |
| | YI-2 | mean | 381.3 | 110.5 | n.d. | n.d. | n.d. | n.d. | n.d. | n.d. | n.d. | n.d. | n.d. | n.d. | n.d. |
| | | SD | 65.1 | 12.6 | \ | \ | \ | \ | \ | \ | \ | \ | \ | \ | \ |
| | J-1 | mean | 2728.6 | 48.6 | n.d. | 384.8 | n.d. | n.d. | 192.1 | 348.5 | n.d. | n.d. | n.d. | 435.6 | n.d. |
| | | SD | 7.9 | 6.5 | \ | 1.6 | \ | \ | 21.2 | 27.4 | \ | \ | \ | 42.8 | \ |
| | J-2 | mean | 559.4 | 47.7 | 51.3 | n.d. | n.d. | n.d. | n.d. | 47.1 | n.d. | n.d. | n.d. | 206.8 | n.d. |
| | | SD | 65.5 | 7.6 | 9.9 | \ | \ | \ | \ | 4.2 | \ | \ | \ | 13.4 | \ |
| | J-3 | mean | 568.8 | 69.6 | n.d. | 423.2 | n.d. | n.d. | n.d. | 92.6 | n.d. | n.d. | n.d. | 264.6 | n.d. |
| | | SD | 17.1 | 9.7 | \ | 11.8 | \ | \ | \ | 4.2 | \ | \ | \ | 9.5 | \ |
| | H-1 | mean | 256.3 | n.q. | n.d. | n.d. | n.d. | n.d. | n.d. | 69.3 | n.d. | n.d. | n.d. | n.q. | n.d. |
| | | SD | 49.6 | \ | \ | \ | \ | \ | \ | 9.9 | \ | \ | \ | \ | \ |
| | H-2 | mean | 183.9 | 27.9 | n.d. | n.d. | n.d. | n.d. | n.d. | 158.6 | n.d. | n.d. | 43.7 | 127.1 | n.d. |
| | | SD | 8.0 | 1.2 | \ | \ | \ | \ | \ | 11.5 | \ | \ | 3.9 | 24.6 | \ |
| | H-3 | mean | 136.8 | n.q. | n.d. | n.d. | n.d. | n.d. | n.d. | 79.2 | n.d. | n.d. | n.d. | n.q. | n.d. |
| | | SD | 1.0 | \ | \ | \ | \ | \ | \ | 2.5 | \ | \ | \ | \ | \ |
| Northeast | JI-1 | mean | 867.4 | 72.7 | 71.4 | n.d. | n.d. | n.d. | 145.7 | 82.9 | 33.8 | n.d. | n.d. | 153.5 | n.d. |
| | | SD | 153.5 | 4.5 | 12.7 | \ | \ | \ | 2.1 | 15.5 | 3.4 | \ | \ | 4.5 | \ |
| | L-1 | mean | 439.9 | 42.9 | 69.1 | n.d. | n.d. | n.d. | 218.1 | 154.8 | n.d. | n.d. | n.d. | 291.4 | n.d. |
| | | SD | 38.3 | 3.5 | 1.4 | \ | \ | \ | 1.0 | 6.4 | \ | \ | \ | 45.2 | \ |
| | L-2 | mean | 719.0 | 46.6 | 51.2 | n.d. | n.d. | n.d. | n.d. | 133.5 | n.d. | n.d. | 72.4 | 233.5 | n.d. |
| | | SD | 41.2 | 3.7 | 9.3 | \ | \ | \ | \ | 13.6 | \ | \ | 8.1 | 8.5 | \ |
| Region | Sample | ng/kg | STZ | TMP | AZI | DANME | DMZ | LOM | MTZ | OFX | RND | TYL | CHL | FFC | TAP |
| | Q-1 | mean | n.d. | n.d. | n.d. | n.d. | 39.2 | 20.8 | n.q. | 212.1 | n.d. | n.d. | n.d. | n.d. | n.d. |
| Northwest | | SD | \ | \ | \ | \ | 7.3 | 2.8 | \ | 27.4 | \ | \ | \ | \ | \ |
| | Q-2 | mean | n.d. | n.d. | n.d. | n.d. | n.d. | n.q. | n.q. | n.q. | n.d. | n.d. | n.d. | n.d. | n.d. |

| | | | | | | | | | | | | | | |
|---|---|---|---|---|---|---|---|---|---|---|---|---|---|---|
| | SD | \ | \ | \ | \ | \ | \ | \ | \ | \ | \ | \ | \ | \ |
| Q-3 | mean | n.d. | n.d. | n.d. | n.d. | n.d. | n.d. | n.q. | 50.3 | n.d. | n.d. | n.d. | n.d. | n.d. |
| | SD | \ | \ | \ | \ | \ | \ | \ | 7.1 | \ | \ | \ | \ | \ |
| X-1 | mean | n.d. | n.d. | n.d. | n.d. | 43.4 | n.d. | n.q. | n.q. | n.d. | n.d. | n.d. | n.d. | n.d. |
| | SD | \ | \ | \ | \ | 6.5 | \ | \ | \ | \ | \ | \ | \ | \ |
| X-2 | mean | n.d. | n.q. | n.q. | n.d. | 60.5 | n.d. | n.q. | 52.4 | n.d. | n.d. | n.d. | n.d. | n.d. |
| | SD | \ | \ | \ | \ | 7.1 | \ | \ | 4.8 | \ | \ | \ | \ | \ |
| X-3 | mean | n.d. | n.d. | n.d. | n.d. | 27.7 | n.d. | n.d. | n.q. | n.d. | n.d. | n.d. | n.d. | n.d. |
| | SD | \ | \ | \ | \ | 3.3 | \ | \ | \ | \ | \ | \ | \ | \ |
| X-4 | mean | n.d. | n.d. | n.d. | n.d. | 38.0 | n.d. | n.q. | n.q. | n.d. | n.d. | n.d. | n.d. | n.d. |
| | SD | \ | \ | \ | \ | 5.1 | \ | \ | \ | \ | \ | \ | \ | \ |
| X-5 | mean | n.d. | n.d. | n.d. | n.d. | n.d. | n.d. | n.d. | n.q. | n.d. | n.d. | n.d. | n.d. | n.d. |
| | SD | \ | \ | \ | \ | \ | \ | \ | \ | \ | \ | \ | \ | \ |
| N-1 | mean | n.d. | n.d. | n.d. | n.d. | 41.2 | n.d. | n.q. | 54.1 | n.d. | n.d. | n.d. | n.d. | n.d. |
| | SD | \ | \ | \ | \ | 1.2 | \ | \ | 3.7 | \ | \ | \ | \ | \ |
| N-2 | mean | n.d. | n.d. | n.d. | n.d. | 33.4 | n.d. | n.q. | n.q. | n.d. | n.d. | n.d. | n.d. | n.d. |
| | SD | \ | \ | \ | \ | 2.1 | \ | \ | \ | \ | \ | \ | \ | \ |
| N-3 | mean | n.d. | n.d. | n.d. | n.d. | n.d. | n.d. | n.q. | n.q. | n.d. | n.d. | n.d. | n.d. | n.d. |
| | SD | \ | \ | \ | \ | \ | \ | \ | \ | \ | \ | \ | \ | \ |
| QIN-1 | mean | n.d. | n.d. | n.d. | 182.2 | n.d. | n.d. | n.q. | 182.3 | n.d. | n.d. | n.d. | n.d. | n.d. |
| | SD | \ | \ | \ | 10.2 | \ | \ | \ | 9.9 | \ | \ | \ | \ | \ |
| QIN-2 | mean | n.d. | n.d. | n.d. | n.d. | n.d. | n.d. | n.q. | 32.6 | n.d. | n.d. | n.d. | n.d. | n.d. |
| | SD | \ | \ | \ | \ | \ | \ | \ | 0.8 | \ | \ | \ | \ | \ |
| QIN-3 | mean | n.d. | n.d. | n.d. | n.d. | 30.6 | n.d. | n.q. | 31.7 | n.q. | n.d. | n.d. | n.d. | n.d. |
| | SD | \ | \ | \ | \ | 5.2 | \ | \ | 1.7 | \ | \ | \ | \ | \ |
| GAN-1 | mean | n.d. | n.d. | n.d. | n.d. | n.q. | n.d. | n.d. | n.q. | n.d. | n.d. | n.d. | n.d. | n.d. |

| | | | | | | | | | | | | | | | |
|---|---|---|---|---|---|---|---|---|---|---|---|---|---|---|---|
| | | SD | \ | \ | \ | \ | \ | \ | \ | \ | \ | \ | \ | \ | \ |
| | GAN-2 | mean | n.d. | n.d. | n.d. | n.d. | n.d. | n.d. | 20.4 | n.q. | n.d. | n.d. | n.d. | n.d. | n.d. |
| | | SD | \ | \ | \ | \ | \ | \ | 0.2 | \ | \ | \ | \ | \ | \ |
| | GAN-3 | mean | n.d. | n.d. | n.d. | n.d. | n.d. | n.d. | 28.8 | 43.3 | n.d. | n.d. | n.d. | n.d. | n.d. |
| | | SD | \ | \ | \ | \ | \ | \ | 0.6 | 7.4 | \ | \ | \ | \ | \ |
| Southwest | YUN-1 | mean | n.d. | 143.1 | n.d. | n.d. | n.d. | 263.2 | 25.8 | 524.6 | n.d. | n.d. | n.d. | 679.3 | n.d. |
| | | SD | \ | 16.8 | \ | \ | \ | 38.3 | 2.3 | 45.8 | \ | \ | \ | 30.4 | \ |
| | YUN-2 | mean | n.d. | n.d. | n.d. | n.d. | n.q. | 156.9 | 34.4 | 148.4 | n.d. | n.d. | n.d. | n.d. | n.d. |
| | | SD | \ | \ | \ | \ | \ | 8.4 | 1.7 | 28.2 | \ | \ | \ | \ | \ |
| | YUN-3 | mean | n.d. | n.d. | n.d. | n.d. | n.q. | n.d. | 46.8 | n.q. | n.d. | n.d. | n.d. | n.d. | n.d. |
| | | SD | \ | \ | \ | \ | \ | \ | 0.6 | \ | \ | \ | \ | \ | \ |
| | YUN-4 | mean | n.d. | n.d. | n.d. | n.d. | n.d. | n.d. | 25.4 | n.q. | n.d. | n.d. | n.d. | n.d. | n.d. |
| | | SD | \ | \ | \ | \ | \ | \ | 1.6 | \ | \ | \ | \ | \ | \ |
| | YU-1 | mean | n.d. | n.d. | n.d. | n.d. | n.d. | n.d. | n.d. | n.q. | n.d. | n.d. | n.d. | n.d. | n.d. |
| | | SD | \ | \ | \ | \ | \ | \ | \ | \ | \ | \ | \ | \ | \ |
| | SHU-1 | mean | n.d. | n.d. | n.d. | n.d. | n.q. | n.d. | n.d. | n.q. | n.d. | n.d. | n.d. | n.d. | n.d. |
| | | SD | \ | \ | \ | \ | \ | \ | \ | \ | \ | \ | \ | \ | \ |
| | G-1 | mean | n.d. | n.d. | n.d. | n.d. | n.q. | n.d. | 20.3 | n.q. | n.d. | n.d. | n.d. | n.d. | n.d. |
| | | SD | \ | \ | \ | \ | \ | \ | 0.3 | \ | \ | \ | \ | \ | \ |
| South China | YUE-1 | mean | 51.0 | 111.7 | n.d. | n.d. | n.q. | n.d. | 49.4 | 415.9 | n.d. | n.d. | 92.4 | 106.6 | n.d. |
| | | SD | 7.5 | 16.9 | \ | \ | \ | \ | 6.1 | 64.4 | \ | \ | 17.5 | 1.4 | \ |
| | GUI-1 | mean | n.d. | n.d. | n.d. | n.d. | n.d. | n.d. | n.d. | n.q. | n.d. | n.d. | n.d. | n.d. | n.d. |
| | | SD | \ | \ | \ | \ | \ | \ | \ | \ | \ | \ | \ | \ | \ |
| | QIONG-1 | mean | n.d. | n.d. | n.d. | n.d. | n.d. | n.d. | 24.0 | n.d. | n.d. | n.d. | n.d. | n.d. | n.d. |
| | | SD | \ | \ | \ | \ | \ | \ | 2.3 | \ | \ | \ | \ | \ | \ |
| Central China | Y-1 | mean | n.d. | n.d. | 434.8 | n.d. | 40.8 | n.d. | n.q. | 104.6 | n.d. | n.d. | n.d. | n.d. | n.d. |

| | | | | | | | | | | | | | | | |
|---|---|---|---|---|---|---|---|---|---|---|---|---|---|---|---|
| | | SD | \ | \ | 41.4 | \ | 1.1 | \ | \ | 5.6 | \ | \ | \ | \ | \ |
| | Y-2 | mean | n.d. | n.d. | n.d. | n.d. | 50.9 | n.d. | n.d. | n.q. | n.d. | n.d. | n.d. | n.d. | n.d. |
| | | SD | \ | \ | \ | \ | 9.5 | \ | \ | \ | \ | \ | \ | \ | \ |
| | Y-3 | mean | n.d. | n.d. | n.d. | n.d. | 75.0 | n.d. | n.q. | n.q. | n.d. | n.d. | n.d. | n.d. | n.d. |
| | | SD | \ | \ | \ | \ | 2.2 | \ | \ | \ | \ | \ | \ | \ | \ |
| | E-1 | mean | n.d. | n.d. | n.d. | n.d. | n.q. | n.d. | n.d. | 27.2 | n.d. | n.d. | n.d. | n.d. | n.d. |
| | | SD | \ | \ | \ | \ | \ | \ | \ | 2.7 | \ | \ | \ | \ | \ |
| | XIANG-1 | mean | n.d. | n.d. | n.d. | n.d. | n.d. | n.d. | n.d. | n.q. | n.d. | n.d. | n.d. | n.d. | n.d. |
| | | SD | \ | \ | \ | \ | \ | \ | \ | \ | \ | \ | \ | \ | \ |
| | M-1 | mean | 51.2 | 55.3 | n.d. | n.d. | n.d. | 445.9 | 55.9 | 103.4 | n.d. | n.d. | n.d. | n.d. | n.d. |
| | | SD | 6.4 | 3.8 | \ | \ | \ | 41.9 | 7.6 | 1.5 | \ | \ | \ | \ | \ |
| | Z-1 | mean | n.d. | n.d. | n.d. | n.d. | n.d. | n.d. | n.d. | n.q. | n.d. | n.d. | n.d. | n.d. | n.d. |
| | | SD | \ | \ | \ | \ | \ | \ | \ | \ | \ | \ | \ | \ | \ |
| | WAN-1 | mean | n.d. | n.d. | n.d. | n.d. | n.q. | n.d. | n.d. | n.q. | n.d. | n.d. | n.d. | n.d. | n.d. |
| | | SD | \ | \ | \ | \ | \ | \ | \ | \ | \ | \ | \ | \ | \ |
| | WAN-2 | mean | n.d. | n.d. | n.d. | n.d. | n.d. | n.d. | n.d. | n.q. | n.d. | n.d. | n.d. | n.d. | n.d. |
| | | SD | \ | \ | \ | \ | \ | \ | \ | \ | \ | \ | \ | \ | \ |
| Eastern China | S-1 | mean | n.d. | n.d. | n.d. | n.d. | n.d. | n.d. | n.d. | n.q. | n.d. | n.d. | n.d. | n.d. | n.d. |
| | | SD | \ | \ | \ | \ | \ | \ | \ | \ | \ | \ | \ | \ | \ |
| | HU-1 | mean | n.d. | n.d. | n.d. | n.d. | n.d. | n.d. | n.d. | n.q. | n.d. | n.d. | n.d. | n.d. | n.d. |
| | | SD | \ | \ | \ | \ | \ | \ | \ | \ | \ | \ | \ | \ | \ |
| | HU-2 | mean | n.d. | n.d. | n.d. | n.d. | n.d. | n.d. | n.d. | n.q. | n.d. | n.d. | n.d. | n.d. | n.d. |
| | | SD | \ | \ | \ | \ | \ | \ | \ | \ | \ | \ | \ | \ | \ |
| | LU-1 | mean | n.d. | n.d. | n.d. | n.d. | 44.2 | n.d. | n.q. | n.q. | n.d. | n.d. | n.d. | n.d. | n.d. |
| | | SD | \ | \ | \ | \ | 3.2 | \ | \ | \ | \ | \ | \ | \ | \ |
| | LU-2 | mean | n.d. | n.d. | n.d. | n.d. | n.d. | n.d. | n.d. | n.q. | n.d. | n.d. | n.d. | n.d. | n.d. |

| | | | | | | | | | | | | | | | |
|---|---|---|---|---|---|---|---|---|---|---|---|---|---|---|---|
| | | SD | \ | \ | \ | \ | \ | \ | \ | \ | \ | \ | \ | \ | \ |
| | LU-3 | mean | n.d. | n.d. | n.d. | n.d. | 80.0 | n.d. | n.d. | n.q. | n.d. | n.d. | n.d. | n.d. | n.d. |
| | | SD | \ | \ | \ | \ | 5.8 | \ | \ | \ | \ | \ | \ | \ | \ |
| | NMG-1 | mean | n.d. | n.d. | n.d. | n.d. | 68.3 | n.q. | n.q. | n.q. | n.d. | n.d. | n.d. | n.d. | n.d. |
| | | SD | \ | \ | \ | \ | 3.7 | \ | \ | \ | \ | \ | \ | \ | \ |
| | NMG-2 | mean | n.d. | n.d. | n.d. | n.d. | 60.8 | n.d. | n.d. | n.q. | n.d. | n.d. | n.d. | n.d. | n.d. |
| | | SD | \ | \ | \ | \ | 4.2 | \ | \ | \ | \ | \ | \ | \ | \ |
| | JIN-1 | mean | n.d. | n.d. | n.d. | n.d. | n.d. | n.d. | 41.3 | 113.0 | n.q. | n.d. | n.d. | n.d. | n.d. |
| | | SD | \ | \ | \ | \ | \ | \ | 0.2 | 21.2 | \ | \ | \ | \ | \ |
| | YI-1 | mean | n.d. | n.d. | n.d. | n.d. | n.d. | n.d. | n.d. | n.q. | n.d. | n.d. | n.d. | n.d. | n.d. |
| North China | | SD | \ | \ | \ | \ | \ | \ | \ | \ | \ | \ | \ | \ | \ |
| | YI-2 | mean | n.d. | n.d. | n.d. | n.d. | n.q. | n.d. | n.d. | n.q. | n.d. | n.d. | n.d. | n.d. | n.d. |
| | | SD | \ | \ | \ | \ | \ | \ | \ | \ | \ | \ | \ | \ | \ |
| | J-1 | mean | n.d. | n.d. | n.d. | n.d. | n.d. | n.d. | n.d. | n.q. | n.d. | n.d. | n.d. | n.d. | n.d. |
| | | SD | \ | \ | \ | \ | \ | \ | \ | \ | \ | \ | \ | \ | \ |
| | J-2 | mean | n.d. | n.d. | n.d. | n.d. | n.q. | n.d. | n.d. | n.q. | n.d. | n.d. | n.d. | n.d. | n.d. |
| | | SD | \ | \ | \ | \ | \ | \ | \ | \ | \ | \ | \ | \ | \ |
| | J-3 | mean | n.d. | n.d. | n.d. | n.d. | n.d. | n.d. | 22.9 | n.q. | n.d. | n.d. | n.d. | n.d. | n.d. |
| | | SD | \ | \ | \ | \ | \ | \ | 0.6 | \ | \ | \ | \ | \ | \ |
| | H-1 | mean | n.d. | n.d. | n.d. | n.d. | n.d. | n.q. | n.q. | n.q. | n.d. | n.d. | n.d. | n.d. | n.d. |
| | | SD | \ | \ | \ | \ | \ | \ | \ | \ | \ | \ | \ | \ | \ |
| | H-2 | mean | n.d. | n.d. | n.d. | n.d. | n.d. | n.q. | n.d. | n.q. | n.d. | n.d. | n.d. | n.d. | n.d. |
| Northeast | | SD | \ | \ | \ | \ | \ | \ | \ | \ | \ | \ | \ | \ | \ |
| | H-3 | mean | n.d. | n.d. | n.d. | n.d. | n.d. | n.d. | n.q. | n.q. | n.d. | n.d. | n.d. | n.d. | n.d. |
| | | SD | \ | \ | \ | \ | \ | \ | \ | \ | \ | \ | \ | \ | \ |
| | JI-1 | mean | n.d. | n.d. | n.d. | n.d. | n.d. | 158.5 | 24.9 | n.q. | n.d. | n.d. | n.d. | n.d. | n.d. |

| | | | | | | | | | | | | | | |
|---|---|---|---|---|---|---|---|---|---|---|---|---|---|---|
| | SD | \ | \ | \ | \ | \ | 3.9 | 4.5 | \ | \ | \ | \ | \ | \ |
| L-1 | mean | n.d. | n.d. | n.d. | n.d. | n.q. | n.d. | n.d. | n.q. | n.d. | n.d. | n.d. | n.d. | n.d. |
| | SD | \ | \ | \ | \ | \ | \ | \ | \ | \ | \ | \ | \ | \ |
| L-2 | mean | n.d. | n.d. | n.d. | n.d. | n.d. | n.d. | 25.6 | 37.8 | n.d. | n.d. | n.d. | n.d. | n.d. |
| | SD | \ | \ | \ | \ | \ | \ | 0.3 | 5.5 | \ | \ | \ | \ | \ |

n.d.: below the LOD; n.q.: below the LOQ.

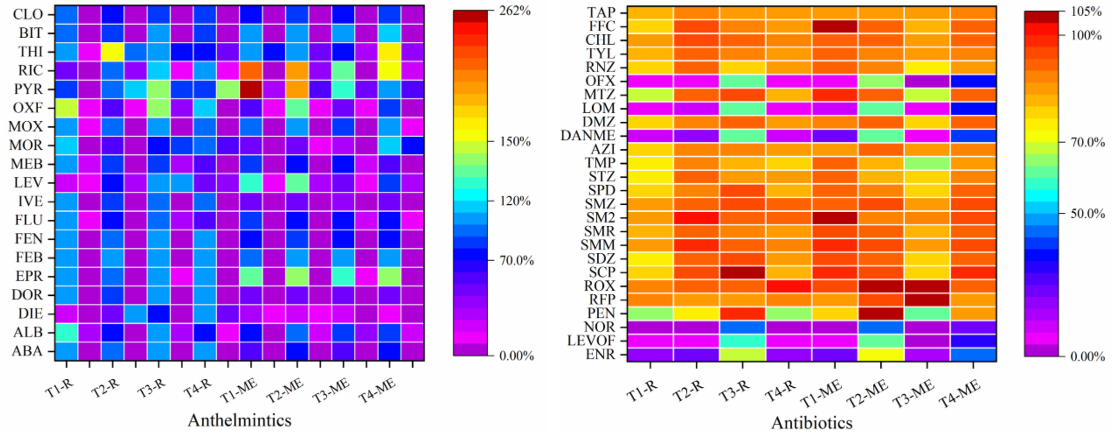


Figure S1 Recoveries (R) and Matrix effects (ME) of the antibiotics and anthelmintics from different extraction versions.

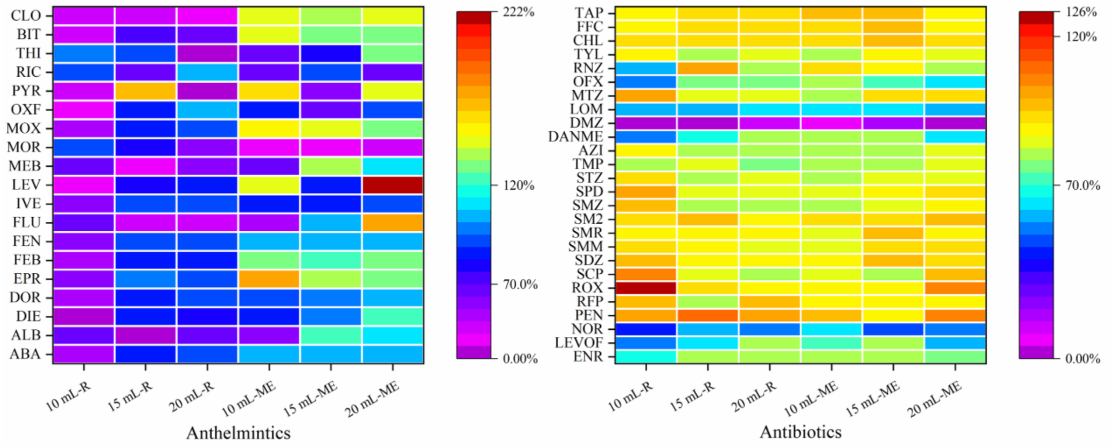


Figure S2 Recoveries (R) and Matrix effects (ME) of the antibiotics and anthelmintics from different volume of extractant.

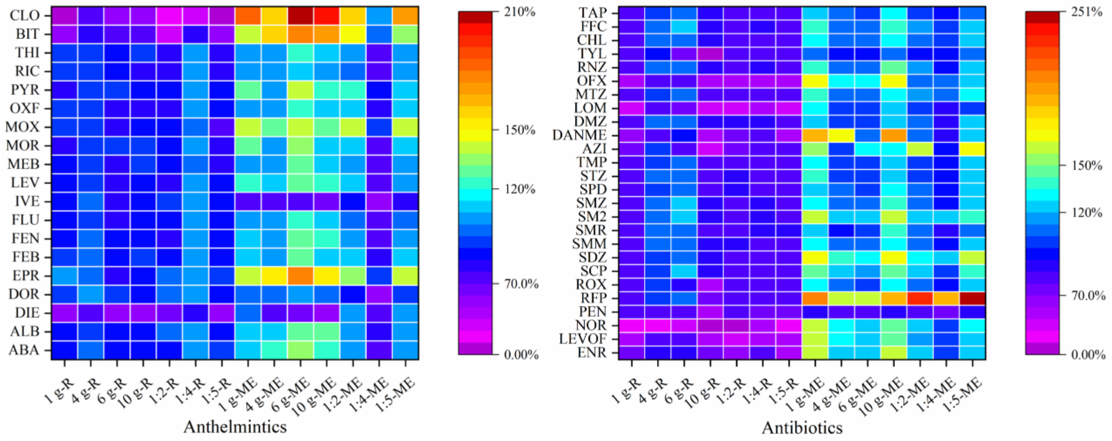


Figure S3 Recoveries (R) and Matrix effects (MEs) of the antibiotics and anthelmintics from different additive amounts of anhydrous MgSO4 for dehydration and different ratio of sodium chloride to anhydrous $MgSO_4$ for buffering.

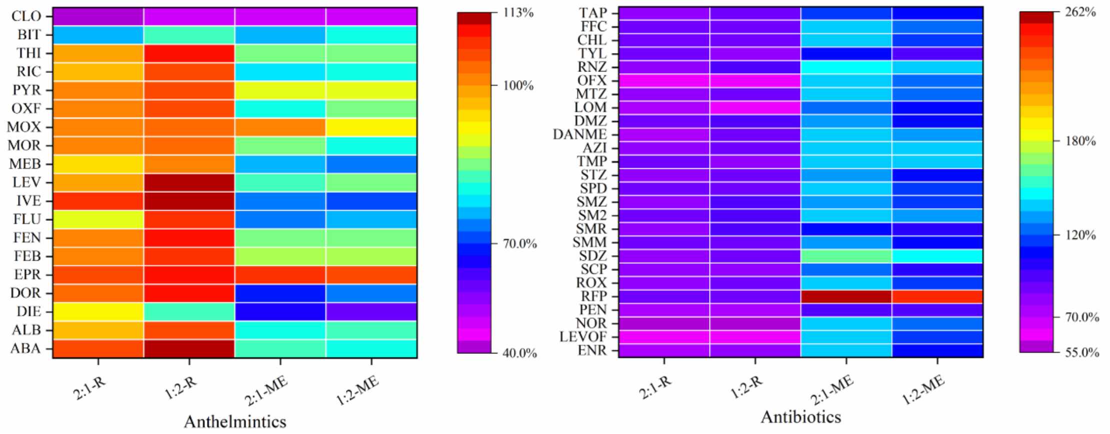


Figure S4 Recoveries (R) and Matrix effects (MEs) of the antibiotics and anthelmintics from different ratio of SCDS to SCTD for buffering.

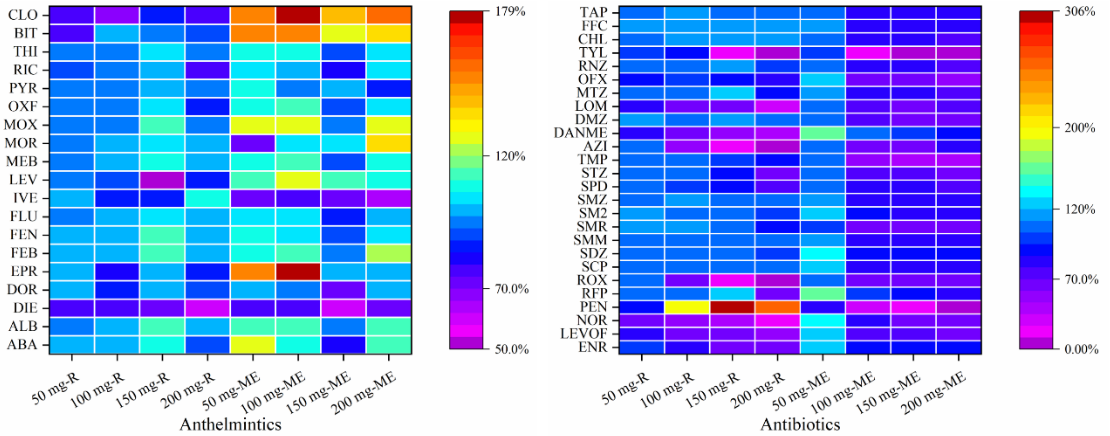


Figure 5 Recoveries (R) and Matrix effects (MEs) of the antibiotics and anthelmintics from different additive amounts of anhydrous MgSO4 for purification.

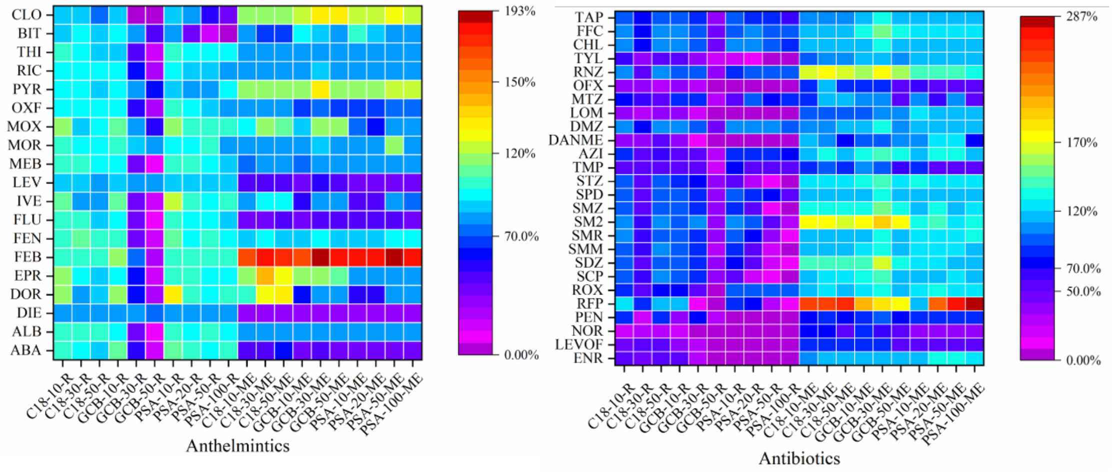


Figure S6 Recoveries (R) and Matrix effects (MEs) of the antibiotics and anthelmintics from different additive amounts of GCB, C18 and PSA for purification.

Reference:

1. MEP. Ministry of environmental protection of the People's republic of China (MEP). *In Exposure Factors Handbook of Chinese Population*; China Environmental Science Press: Beijing, China, 2013.

2. MEP. Ministry of environmental protection of the People's republic of China (MEP). *In Exposure Factors Handbook of Chinese Population* (6~17 years) ; China Environmental Science Press: Beijing, China, 2015a.

3. MEP. Ministry of environmental protection of the People's republic of China (MEP). *In Exposure Factors Handbook of Chinese Population* (0~5 years) ; China Environmental Science Press: Beijing, China, 2015b.

4. Zhai F. *A Prospective Study on Dietary Pattern and Nutrition Transition in China ;* Science Press: Beijing, China, 2008.
